# PAX6 Isoforms, along with Reprogramming Factors, Differentially Regulate the Induction of Cornea-specific Genes

**DOI:** 10.1038/srep20807

**Published:** 2016-02-22

**Authors:** Yuzuru Sasamoto, Ryuhei Hayashi, Sung-Joon Park, Mihoko Saito-Adachi, Yutaka Suzuki, Satoshi Kawasaki, Andrew J. Quantock, Kenta Nakai, Motokazu Tsujikawa, Kohji Nishida

**Affiliations:** 1 Department of Ophthalmology, Osaka University Medical School, Suita, Osaka 565-0871, Japan; 2 Department of Stem Cell and Applied Medicine, Osaka University Medical School, Suita, Osaka 565-0871, Japan; 3 Laboratory of Functional Analysis in silico, The Institute of Medical Science, The University of Tokyo, Minato-ku, Tokyo 108-8639, Japan; 4 Division of Cancer Genomics, National Cancer Center Research Institute, Chuo-ku, Tokyo 104-0045, Japan; 5 Department of Computational Biology, Graduate School of Frontier Sciences, The University of Tokyo, Kashiwa, Chiba 277-8568, Japan; 6 Structural Biophysics Group, School of Optometry and Vision Sciences, Cardiff University, Cardiff CF24 4HQ, Wales, United Kingdom

## Abstract

PAX6 is the key transcription factor involved in eye development in humans, but the differential functions of the two PAX6 isoforms, isoform-a and isoform-b, are largely unknown. To reveal their function in the corneal epithelium, PAX6 isoforms, along with reprogramming factors, were transduced into human non-ocular epithelial cells. Herein, we show that the two PAX6 isoforms differentially and cooperatively regulate the expression of genes specific to the structure and functions of the corneal epithelium, particularly keratin 3 (KRT3) and keratin 12 (KRT12). PAX6 isoform-a induced KRT3 expression by targeting its upstream region. KLF4 enhanced this induction. A combination of PAX6 isoform-b, KLF4, and OCT4 induced KRT12 expression. These new findings will contribute to furthering the understanding of the molecular basis of the corneal epithelium specific phenotype.

PAX6 is the key transcription factor for the development of the eye in humans^1^. PAX6 has two DNA-binding domains: a paired-domain (PD) and a homeodomain (HD)^2^. PD contains an N-terminal sub-domain (PAI domain) and a C-terminal sub-domain (RED domain). Structural variations in the PAI domain define two major PAX6 isoforms, isoform-a (PAX6-a) and isoform-b (PAX6-b). The key difference is that the PAI domain of PAX6-b possesses an extra exon 5a^1^,^3^,^4^. Such a structural variation leads to unique DNA-binding properties. In fact, PAX6-a and PAX6-b present a variety of target genes^3^,^5^,^6^. It is known that the two PAX6 isoforms cooperatively act in the development of the posterior segment of the eye in humans^7^. PAX6 is also known to be essential for the development and maintenance of the anterior segment of the eye, including the corneal epithelium, which envelops the entire optical surface of the eye^8^,^9^,^10^. However, the function of the two PAX6 isoforms in the corneal epithelium is still largely unknown^11^.

To address this question, we transduced the two PAX6 isoforms into the human oral mucosal epithelium, which is used for the reconstruction of the ocular surface in cases of severe corneal epithelial defect but lacks the corneal epithelial phenotype^12^,^13^, and investigated their roles in gene expression and regulation. We particularly focused on corneal epithelium-specific genes, keratin 3 (KRT3) and keratin 12 (KRT12), which are mostly specific to the structure and function of the corneal epithelium^14^,^15^,^16^. We also examined the effect of the Yamanaka factors (OCT4, SOX2, KLF4, and c-Myc), which are known to reprogram cell fate^17^,^18^. Our results reveal that the two PAX6 isoforms differentially and cooperatively regulate the corneal epithelium-specific genes as well as many other genes, and OCT4 and KLF4 enhance their expression.

## Results

### PAX6 is a key factor involved in the corneal epithelial phenotype

**Figure 1:**
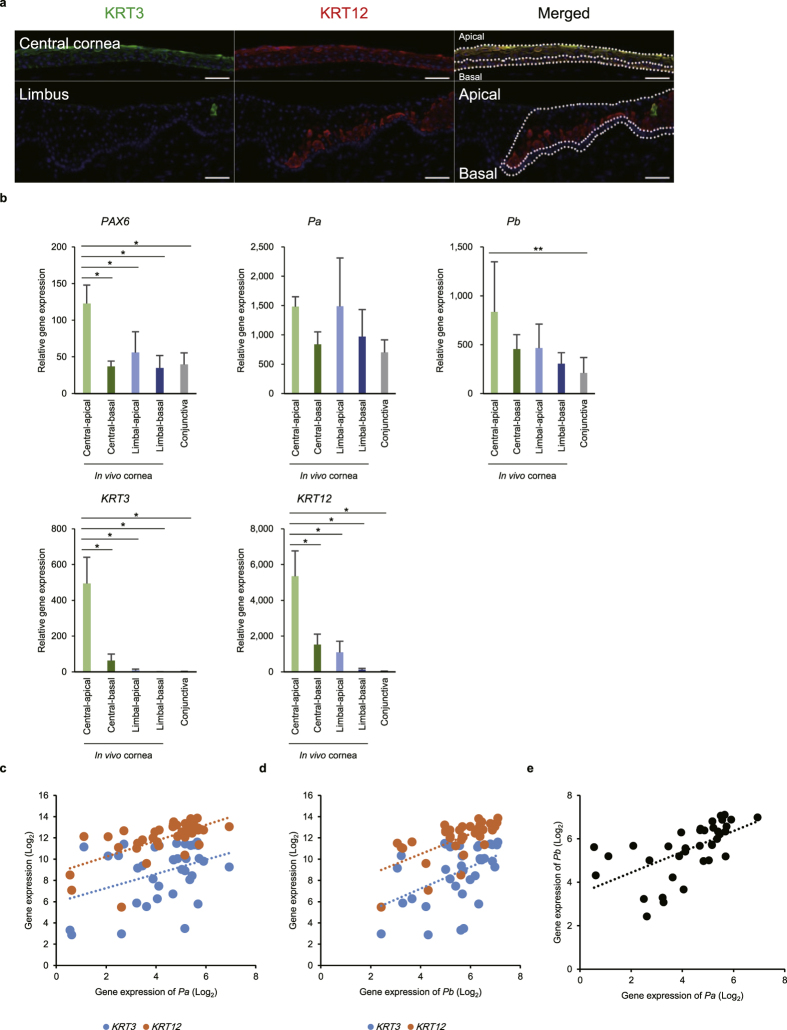
Marker gene expression in the corneal epithelium. (**a**) Immunofluorescence staining of KRT3 and KRT12 in human corneal epithelium *in vivo*. The dotted lines indicate where the micro-dissections were performed. Each scale bar represents 50 μm. (**b**) Quantitative RT-PCR (qRT-PCR) analysis of total *PAX6*, *PAX6-a*, *PAX6-b*, *KRT3* and *KRT12* mRNA levels in 4 areas of the human corneal epithelium (central-apical, central-basal, limbal-apical and limbal-basal) and in the conjunctival epithelium (n = 4). The data are presented as the mean ± standard deviation (SD). *p < 0.01 and **p < 0.05 versus central-apical corneal epithelium by Dunnett’s test. (**c**) Correlation between gene expression levels of *PAX6-a* and *KRT3*, or *KRT12*, in human limbal epithelial cells *in vivo* (n = 37), assessed by a single-cell gene expression analysis (correlation coefficient (r) = 0.38, *p* = 0.02 and r = 0.64, *p* < 0.01, respectively). (**d**) Correlation between the gene expression levels of *PAX6-b* and *KRT3*, or *KRT12*, in human limbal epithelial cells *in vivo* (n = 37), assessed by single-cell gene expression analysis (r = 0.47, *p* < 0.01 and r = 0.64, *p* < 0.01, respectively). (**e**) Correlation between the gene expression levels of *PAX6-a* and *PAX6-b* in human limbal epithelial cells *in vivo* (n = 37), assessed by single-cell gene expression analysis (r = 0.60, *p* < 0.01). Pa, PAX6-isoform-a; Pb, PAX6-isoform-b.

A transcriptome analysis (RNA-seq) of the corneal epithelium and oral mucosal epithelium from mouse embryos confirmed that *Pax6* was relatively highly expressed in the corneal epithelium (Supplementary Fig. S1a–c and Supplementary Table S1), suggesting the key role of *Pax6* in the development of this cell layer. A laser micro-dissection of frozen sections of the human corneal epithelium *in vivo*, separated into four areas (central-apical, central-basal, limbal-apical and limbal-basal) (Fig. 1a), revealed that *PAX6* was present in all areas of the human corneal epithelium, with relatively high expression in the central**-**apical region (Fig. 1b and Supplementary Fig. S1d). Moreover, *PAX6-a* and *PAX6-b* were expressed in all epithelia areas at various levels. The two corneal epithelium-specific keratins, *KRT3* and *KRT12*, were highly expressed in the central**-**apical corneal epithelium, but less so in the other areas (Fig. 1a,b). Single-cell gene expression analysis revealed a positive correlation between both *PAX6* isoforms and *KRT3* and *KRT12* in human limbal epithelial cells *in vivo* (Fig. 1c,d). Moreover, *PAX6-a* and *PAX6-b* were co-expressed in individual cells, as evidenced by a positive correlation of the expression data (correlation coefficient (r) = 0.60, *p* < 0.01) (Fig. 1e).

### PAX6 isoforms, combined with reprogramming factors, differentially regulate KRT3 and KRT12 expression

**Figure 2:**
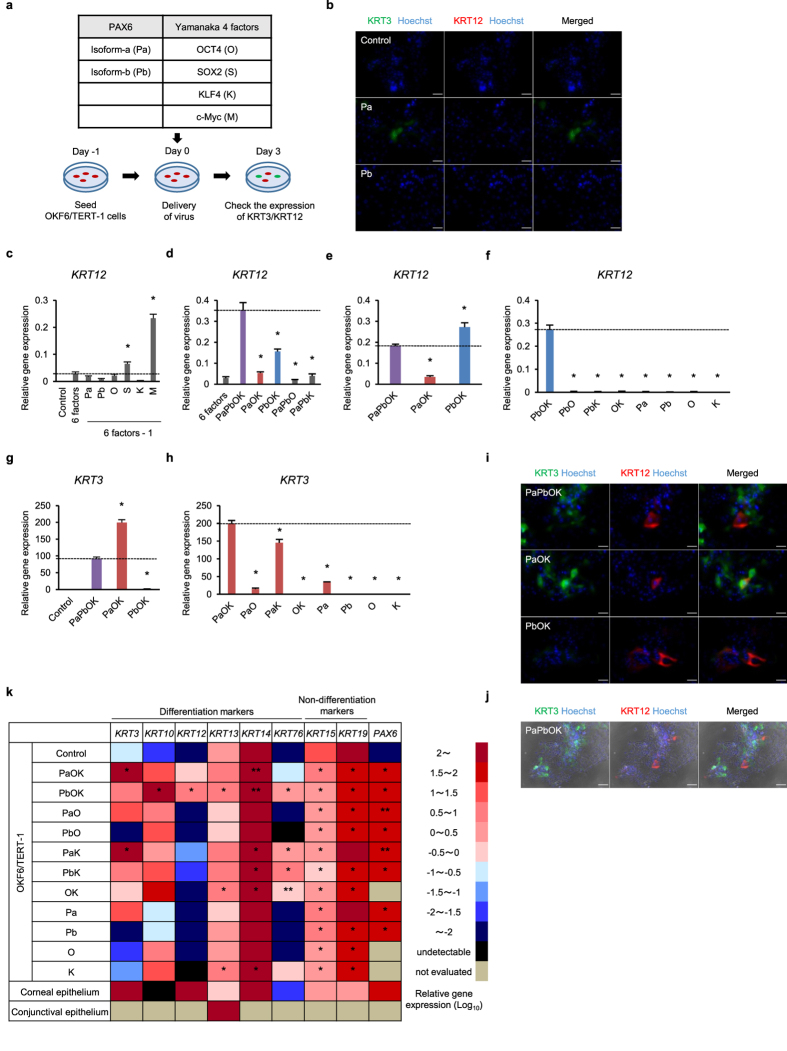
Screening of KRT12 and KRT3 expression levels. (**a**) Schematic representation of the transcription factor screening. (**b**) Immunofluorescence staining of KRT3 and KRT12 in PAX6-a- and PAX6-b-transduced OKF6/TERT-1 cells. (**c**-**f**) qRT-PCR of *KRT12* mRNA levels at day 3 (n = 6). (**g**,**h**) qRT-PCR of *KRT3* mRNA levels at day 3 (n = 6). (**i**) Immunofluorescence staining of KRT12 and KRT3 in PAX6-a-PAX6-b-OCT4-KLF4-, PAX6-a-OCT4-KLF4- and PAX6-b-OCT4-KLF4-transduced OKF6/TERT-1 cells. (**j**) Low-power field of immunofluorescence staining of PAX6-a-PAX6-b-OCT4-KLF4-transduced OKF6/TERT-1 cells laid over a phase contrast image. (**k**) Effect of various patterns of transduction on keratin mRNA levels measured by qRT-PCR after adjusting the total *PAX6* mRNA level in the transduced OKF6/TERT-1 cells to match that of the *in vivo* corneal epithelium (n = 4 to 8). The scale numbers are presented as the log_10_ of the relative gene expression. Human corneal epithelium and conjunctival epithelium *in vivo* were used as controls. Pa, PAX6-isoform-a; Pb, PAX6-isoform-b; O, OCT4; S, SOX2; K, KLF4; M, c-Myc. The data presented in (**c**,**d)**, (**e**,**f)**, and (**g**,**h)** are from the same experiments. The data are presented as the mean ± SEM (**c**–**h**). *p < 0.01 and **p < 0.05 versus control by Dunnett’s test. Scale bars represent 50 μm (**b**,**i**) and 100 μm (**j**).

Owing to the fact that both PAX6-a and PAX6-b were expressed in limbal epithelial cells (Fig. 1e), it was not possible to delineate the regulatory specificity of each isoform. To clarify this, we transduced PAX6-a or PAX6-b into the immortalized oral mucosal epithelial cell line OKF6/TERT-1^19^, which does not express PAX6, and evaluated KRT3 and KRT12 expression (Fig. 2a). PAX6-a induced KRT3 expression, whereas PAX6-a or PAX6-b did not induce KRT12 expression (Fig. 2b). Based on the proposed usefulness of the Yamanaka factors (OCT4, SOX2, KLF4, and c-Myc) in reprogramming cell fate^17^,^18^,^20^,^21^, we investigated how the co-transduction of all four of these factors might affect the cells’ ability to induce KRT12 expression (Fig. 2a). When all six factors (PAX6-a, PAX6-b, and the four Yamanaka factors) were transduced simultaneously, a small amount of *KRT12* expression was detected (Fig. 2c). To further probe which of the six factors were important for *KRT12* induction, they were removed one by one from the cocktail. The absence of either SOX2 or c-Myc resulted in a significantly enhanced level of *KRT12* expression (Fig. 2c). Notably, when both SOX2 and c-Myc were not present, *KRT12* expression significantly increased (Fig. 2d). However, the subsequent removal of one of the four factors (PAX6-a, PAX6-b, OCT4, or KLF4) reduced *KRT12* expression level (Fig. 2d).

*PAX6* expression level is known to be important for eye development^22^. To investigate this, we doubled the amount of one of the PAX6 isoforms and removed the other. Remarkably, the combination of PAX6-b with OCT4 and KLF4 induced *KRT12* expression at higher levels than when both PAX6 isoforms were used together, along with OCT4 and KLF4 (Fig. 2e). The removal of these three factors, alone or in combination, resulted in considerably lower *KRT12* expression levels (Fig. 2f).

Next, we examined the induction of *KRT3* expression and found that PAX6-a, PAX6-b, OCT4, and KLF4 transduction into OKF6/TERT-1 cells significantly increased *KRT3* expression level (Fig. 2g). By selectively removing one isoform and doubling the amount of the other, we confirmed that the absence of PAX6-b enhanced *KRT3* expression (Fig. 2g). Thus, PAX6-a was deemed to be the critical factor for the induction of *KRT3* expression. The experiments also revealed that KLF4, combined with PAX6-a, had a large impact on the regulation of *KRT3* expression (Fig. 2h). Taken together, the combination of PAX6-b-OCT4-KLF4 (for *KRT12*) and either PAX6-a or PAX6-a-KLF4 (for *KRT3*) efficiently induced the corneal epithelium-specific keratin expression in OKF6/TERT-1 cells. The impact of OCT4 on *KRT3* induction was notably less pronounced than its impact on *KRT12* induction. Immunofluorescence staining further showed that KRT12- and/or KRT3-positive cells were relatively strongly stained and were more frequently detected in the regions of the culture where the cells were densely aggregated (Fig. 2i,j and Supplementary Fig. S2a). As such, they were reminiscent of terminally differentiated stratified epithelia. Other than the corneal epithelium-specific keratins, the transgene combinations did not regulate the differentiation markers of other tissues, such as pancreatic islet cells, neurons, the retinal pigment epithelium (RPE), or the lens, in which PAX6 is essential for normal development (Supplementary Fig. S2b).

### KLF4 enhances the expression of keratins

We assessed the expression of *OCT4* and *KLF4* in the human corneal epithelium, and discovered that, whereas *OCT4* was only sparsely expressed throughout the epithelial multiple layers, *KLF4* exhibited relatively high expression levels, especially in the central-apical corneal epithelium where the cells are highly differentiated (Supplementary Fig. S2c,d). Single-cell gene expression analysis showed that *KLF4* expression was positively correlated with *KRT3* and *KRT12* expression in limbal epithelial cells *in vivo* (Supplementary Fig. S2e). In addition, *KRT12* expression in the mouse embryonic corneal epithelium (i.e., murine *Krt12*) increased dramatically at E18.5, which suggests the differentiation of the corneal epithelium, under the influence of high expression levels of *Pax6* and *Klf4* (Supplementary Fig. S2f). The expression levels of other non-corneal epithelium-specific keratins were also altered by different combinations of the transduced factors (Fig. 2k). In particular, KLF4 had a large impact on the up-regulation of the differentiation marker keratins, *KRT3*, *KRT10*, *KRT12*, *KRT13*, *KRT14*, and *KRT76*.

### The region upstream of the *KRT3* gene is a target of PAX6-isoform-a transduction

**Figure 3:**
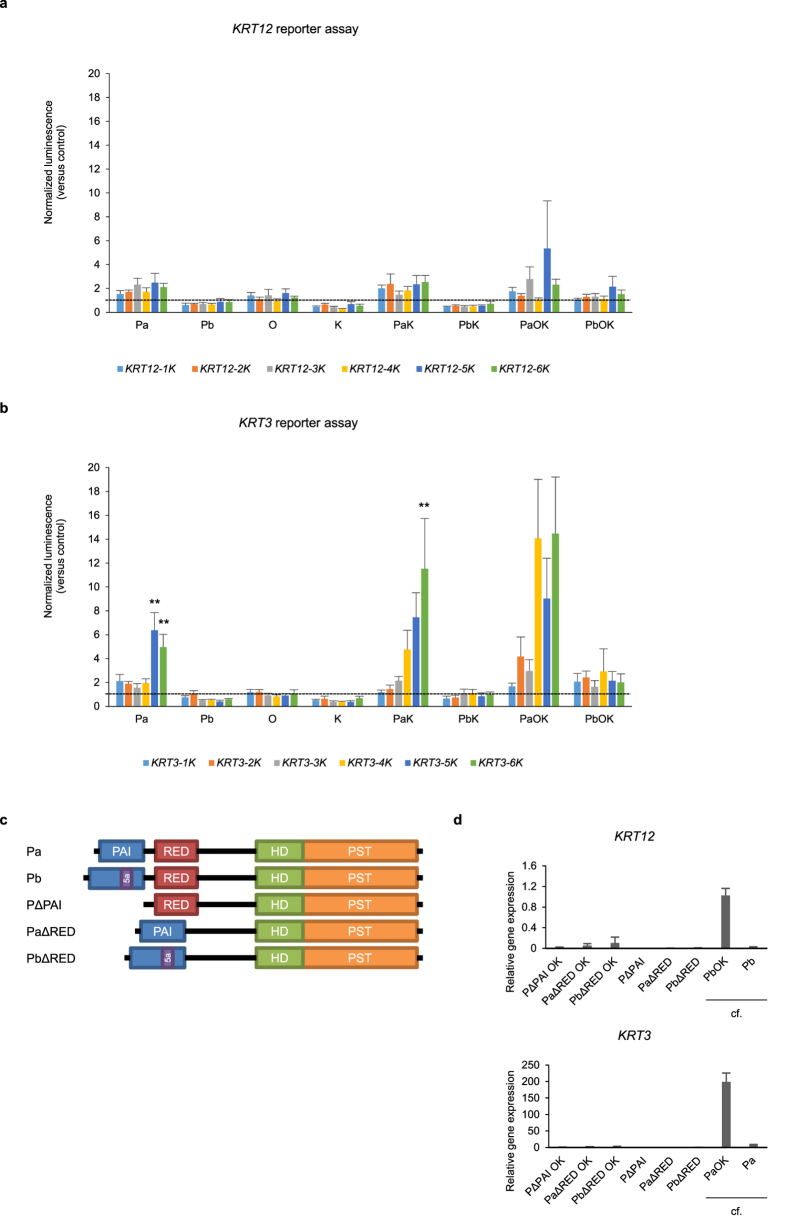
Regulation of KRT12 and KRT3 by two isoforms of PAX6. (**a**,**b**) Luciferase reporter assay using 1 to 6 K base pairs upstream of *KRT12* (**a**) and *KRT3* (**b**). The reporters were co-transfected with the PAX6-a, PAX6-b, OCT4 and KLF4 vectors or their combinations (n = 6). The luminescence was normalized to that of the samples co-transfected with lacZ. **p < 0.05 versus control by paired t-test with a Bonferroni correction. (**c**) Schematic representation of the truncated PAX6 mutants. (**d**) qRT-PCR of *KRT12* and *KRT3* mRNA levels in OKF6/TERT-1 cells transduced with the truncated PAX6 mutants, OCT4 and KLF4 (n = 4 to 9). The mRNA levels of cells transduced with full-length PAX6 from Fig. 2k are included for reference. Pa, PAX6-isoform-a; Pb, PAX6-isoform-b; O, OCT4; K, KLF4; PAI, PAI domain; RED, RED domain; HD, homeodomain; PST, proline/serine/threonine-rich transactivation domain. The data are presented as the mean ± SEM (**a**,**b**,**d**).

Reporter assays were conducted to examine the transcriptional activity of PAX6 isoforms, OCT4, and KLF4 on the expression of *KRT12* and *KRT3*. The *KRT12* reporters did not significantly respond to any overexpression conditions, whereas the *KRT3* reporters responded to the overexpression of PAX6-a, PAX6-a-KLF4, and PAX6-a-OCT4-KLF4 (Fig. 3a,b). Truncated PAX6 mutants (PAX6ΔPAI, PAX6-a-ΔRED, and PAX6-bΔRED), even when combined with OCT4 and KLF4, induced *KRT12* and *KRT3* expression with a low efficiency (Fig. 3c,d), suggesting that both the PAI and RED domains are necessary for the induction of *KRT12* and *KRT3* expression. Furthermore, co-immunoprecipitation followed by mass spectrometry showed that PAX6 did not form protein-protein complexes with the co-transduced factors OCT4 and KLF4 (Supplementary Table S2). These results suggest that PAX6 isoforms bind to their targets via both the PAI and RED domains without forming a complex with OCT4 and KLF4, and that the region upstream of the *KRT3* gene is a target of PAX6-a transduction.

### PAX6 isoforms regulate different genes by forming a highly complex regulatory network in OKF6/TERT-1 cells

**Figure 4:**
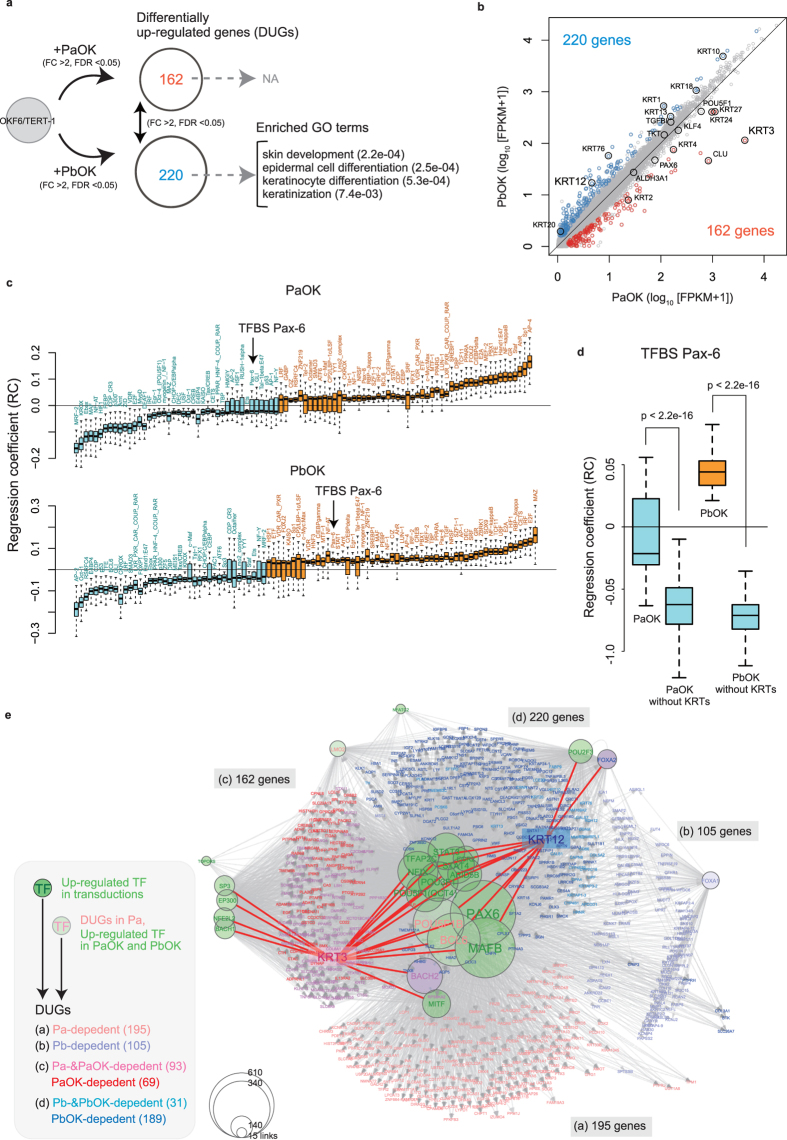
Transcriptome analysis and inference of the regulatory network in OKF6/TERT-1 cells. (**a**) Schematic representation of the identification of DUGs in PAX6-a-OCT4-KLF4 and PAX6-b-OCT4-KLF4 transduced cells. The numbers in parentheses represent the *p*-values of a hypergeometric test with a Bonferroni correction. (**b**) Correlation between the expression levels of PAX6-a-OCT4-KLF4- and PAX6-b-OCT4-KLF4-dependent DUGs. (**c**) Distribution of regression coefficients for 103 putative TFBSs that were considered important to explain the expression levels of PAX6-a-OCT4-KLF4- (upper panel) and PAX6-b-OCT4-KLF4-dependent DUGs (lower panel). TFBS Pax-6 is a putative binding site for PAX6 isoforms. (**d**) Changes in the regression coefficients of TFBS Pax-6 in the absence and presence of keratins (KRTs). (**e**) Gene regulatory network inferred from the network of PAX6-a-OCT4-KLF4 and PAX6-b-OCT4-KLF4 (Supplementary Fig.S3b) and PAX6-a and PAX6-b transductions (Supplementary Fig. S3e). The size of the circles representing the transcription factors in the network is proportional to the total number of targeted genes. Pa, PAX6-isoform-a; Pb, PAX6-isoform-b; O, OCT4; K, KLF4; FC, fold change; FDR, false discovery rate; DUG, differentially up-regulated gene; GO, gene ontology; NA; not available; FPKM, fragments per kilobase of exon per million mapped reads; TF, transcription factor; TFBS, TF-binding site; KRT, keratin. The data are presented as quantile plots (**c**,**d**).

We performed RNA-seq analyses with transduced OKF6/TERT-1 cells and detected 16,725 RefSeq-coding genes that were expressed in at least one cell type. Among these, 1,570 genes were significantly up-regulated in the transduced cells as compared to control cells (>2 fold change [FC], <0.05 false discovery rate [FDR]), 875 genes were up-regulated by both PAX6-a-OCT4-KLF4 and PAX6-b-OCT4-KLF4 transductions, and 695 genes were uniquely up-regulated by each of the transductions (Supplementary Fig. S3a). Remarkably, 24% of the up-regulated genes (382/1,570) were differentially regulated by each of the transductions (>2 FC, Supplementary Table S3), which suggests a regulatory specificity of PAX6 isoforms, 162 by PAX6-a-OCT4-KLF4 transduction and 220 by PAX6-b-OCT4-KLF4 transduction (Fig. 4a). Interestingly, the 220 PAX6-b-OCT4-KLF4-dependent genes were involved in biological processes related to keratinocyte development and differentiation (Fig. 4a) and various keratins were differentially up-regulated (Fig. 4b), which is consistent with the results of the transduction experiments reported in Fig. 2k.

To infer the activity of potential key transcription factors that contribute to the regulation of differentially up-regulated genes (DUGs), we used a linear regression model with putative transcription factor-binding sites (TFBSs) of TRANSFAC^23^ found from DUG promoters. By following a promoter regression modelling^24^, we exhaustively searched for the best combination of TFBSs that explains the expression levels of DUGs. In our dataset, we found 103 TFBSs in total, consisting of 45 identical and 40 opposite mean regression coefficients (RCs) in the transductions (Fig. 4c), of which nine were unique to PAX6-a-OCT4-KLF4- and nine to PAX6-b-OCT4-KLF4-dependent DUGs (Supplementary Table S4). Specifically, TFBSs for Pax-6 bound by PAX6-b presented positive RCs, whereas those bound by PAX6-a could be positive or negative, reflective of the broader transcription activities of PAX6-a. Pax-6 activities were dramatically altered to display negative RCs when keratins were removed from DUGs (Fig. 4d), implying that PAX6 isoforms regulate keratins in the manner of activators.

To visualize the interactions between the potential regulators and DUGs, we built a network based on the results of the promoter modelling and information from a database. We first prepared TRANSFAC transcription factors that are known to bind to TFBSs listed in Fig. 4c. Of these, we further narrowed down to 22 transcription factors, whose coding genes were categorized into 1,570 up-regulated genes. We then linked the 22 transcription factors to 382 DUGs that present binding sites for these transcription factors (Supplementary Fig. S3b and Supplementary Table S5). This network, consisting of 5,170 links, revealed that, although a few transcription factors potentially could regulate either PAX6-a-OCT4-KLF4- or PAX6-b-OCT4-KLF4-dependent DUGs, the majority of these factors shared targeting genes. In particular, PAX6 was connected with all DUGs, and POU5F1 (i.e., OCT4) was linked to 356 out of 382 genes. In addition, we conducted this analysis using the OKF6/TERT-1 cells that had been only transduced with PAX6-a or PAX6-b (Supplementary Fig. S3c,d). We found that eight transcription factors, including PAX6, contributed to the regulation of 424 PAX6-a- and PAX6-b-dependent DUGs (Supplementary Fig. S3e and Supplementary Table S5), which are not likely to be involved in keratinocyte development and differentiation (Supplementary Fig. S3c). These results highlight the importance of OCT4- and KLF4-binding in mediating the regulatory circuit, whereby PAX6 isoforms appear to activate the development and differentiation of the epithelia in general. Overall, the merged network of Supplementary Fig. S3b and Supplementary Fig. S3e, consisting of 6,631 links among 24 up-regulated transcription factors and 669 DUGs, shows the highly complex interactions that include potential key regulators such as broadly connected transcription factors (e.g. PAX6, MAFB, BCL6, and POU5F1B) as well as more specialized factors (e.g. TOPORS, BACH1, FOXA2, and POU2F3) (Fig. 4e and Supplementary Table S5).

### KRT12 induction requires high expression of transgenes

**Figure 5:**
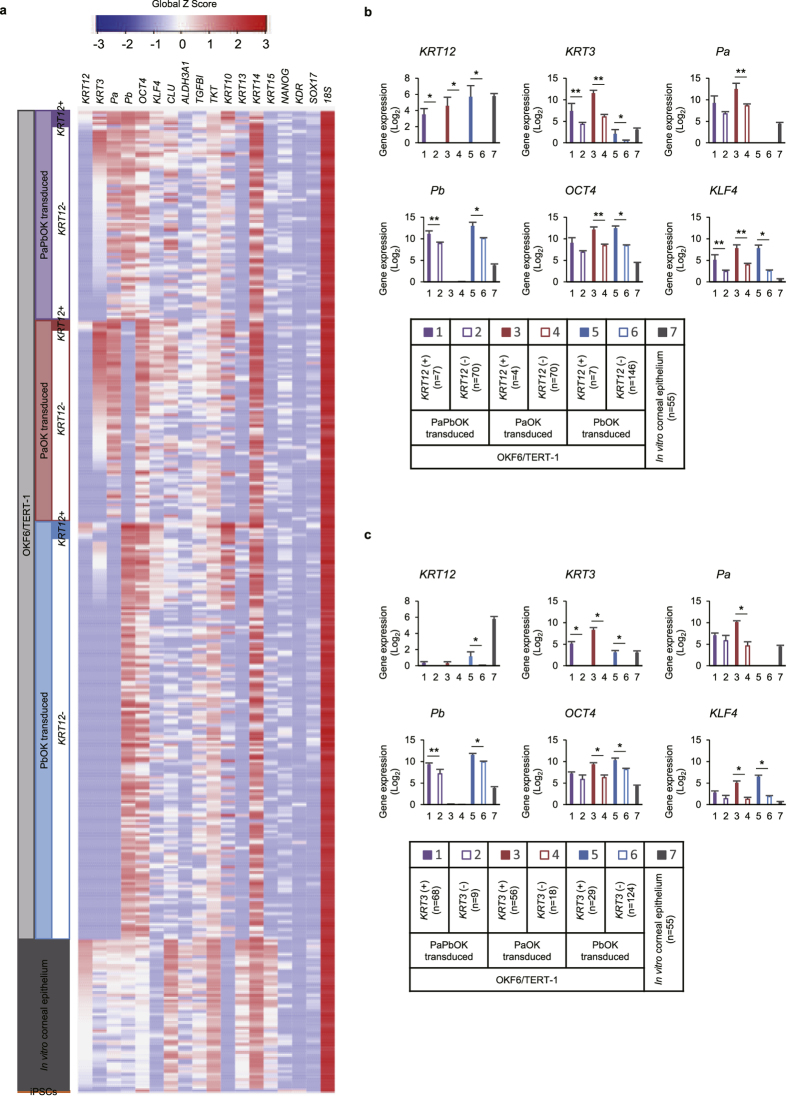
Single-cell gene expression analysis of transduced OKF6/TERT-1 cells. (**a**) Heatmap of the expression (Global Z-Score) of each single OKF6/TERT-1 cell, transduced with PAX6-a-PAX6-b-OCT4-KLF4, PAX6-a-OCT4-KLF4 and PAX6-b-OCT4-KLF4. Corneal epithelial cells *in vitro* and feeder-free iPSCs are listed for reference. (**b**) Gene expression of *KRT12*, *KRT3*, and transgenes, subgrouped by *KRT12* expression level (positive or negative). (**c**) Gene expression of *KRT12*, *KRT3*, and transgenes, subgrouped by *KRT3* expression level (positive or negative). Pa, PAX6-isoform-a; Pb, PAX6-isoform-b; O, OCT4; K, KLF4; iPSCs, induced pluripotent stem cells. The data are presented as the mean ± SEM (**b**,**c**). *p < 0.01 and **p < 0.05 obtained with a t-test.

To elucidate the precise expression pattern of transduced OKF6/TERT-1 cells, we performed single-cell gene expression analysis (Fig. 5a). Individual *KRT12*-positive cells expressed this keratin as highly as that observed in the corneal epithelium *in vitro* (Fig. 5b). Our experiments show that more *KRT3*-positive cells than *KRT12*-positive cells were induced, suggesting that *KRT3* induction was more readily achieved (Fig. 5c). *KRT12-* and *KRT3-*positive cells expressed *PAX6-a*, *PAX6-b*, *OCT4,* and *KLF4* at higher levels than *KRT12*- and *KRT3-*negative cells (Fig. 5b,c), suggesting that the efficient transduction of transgenes supports the induction of *KRT12* and *KRT3*.

The other expressed genes are shown in Supplementary Fig. S4a. *KRT3*-positive cells also expressed clusterin (*CLU*), one of the most abundant genes in the corneal epithelium^25^. *CLU* expression was positively correlated with *PAX6-a* (r = 0.568, *p* < 0.01), but not with *PAX6-b* (r = −0.101, *p* = 0.08) (Supplementary Fig. S4b), consistent with the up-regulation by PAX6-a-OCT4-KLF4 shown by RNA-seq (Fig. 4b and Supplementary Table S3). Among the other genes that were abundant in the corneal epithelium, *ALDH3A1* and *TKT* were also up-regulated under certain conditions (Supplementary Fig. S4a). *KRT12-* and/or *KRT3*-positive cells also tended to express higher levels of the differentiation markers *KRT10*, *KRT13,* and *KRT14* (Supplementary Fig. S4a).

Next, we established the tetracycline on (Tet-On) system for the controlled expression of PAX6-a-OCT4-KLF4 and PAX6-b-OCT4-KLF4 in OKF6/TERT-1 cells using all-in-one vectors. PAX6-a-OCT4-KLF4-induced colonies showed low *KRT12* expression levels, but very high *KRT3* levels following the addition of tetracycline (Tet+) (Supplementary Fig. S4c,d). PAX6-b-OCT4-KLF4-induced colonies, on the other hand, showed very low *KRT12* levels following the addition of tetracycline (Tet+) (Supplementary Fig. S4d,e). This was inconsistent with the finding of *KRT12* induction following PAX6-b-OCT4-KLF4 transduction. However, transduction of PAX6-a-OCT4-KLF4 and PAX6-b-OCT4-KLF4 in these PAX6-b-OCT4-KLF4-inducible colonies, in the absence of tetracycline (Tet−), induced *KRT12* expression (Supplementary Fig. S4d,e). This suggests that a high expression level of PAX6-b-OCT4-KLF4 is required for *KRT12* expression, consistent with the results of the single-cell gene expression analysis (Fig. 5b).

### Effects of the epigenetic state on KRT12 induction

**Figure 6:**
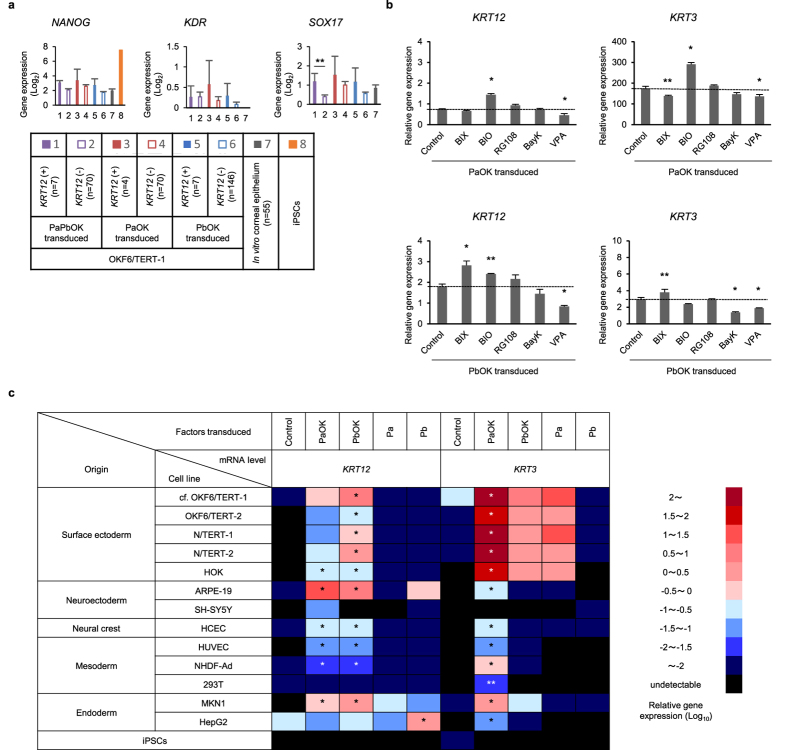
Effects of the epigenetic state on KRT12 induction and of transgenes on different cell types. (**a**) Gene expression of *NANOG*, *KDR*, and *SOX17*, subgrouped by *KRT12* expression level (positive or negative). **p < 0.05 by t-test. (**b**) qRT-PCT analysis of *KRT12* and *KRT3* mRNA levels in PAX6-a-OCT4-KLF4- or PAX6-b-OCT4-KLF4-transduced OKF6/TERT-1 cells, incubated with small molecules (n = 4). *p < 0.01 and **p < 0.05 versus control, obtained by a Dunnett’s test. (**c**) qRT-PCR analysis of *KRT12* and *KRT3* mRNA levels in different human cells (n = 4 to 8) transduced with PAX6-a, PAX6-b, OCT4, and KLF4. The scale numbers are presented as the log_10_ of the relative gene expression. The mRNA levels of transduced OKF6/TERT-1 cells from Fig. 2k are listed for reference. *p < 0.01 and **p < 0.05 versus control, obtained by a Dunnett’s test. Pa, PAX6-isoform-a; Pb, PAX6-isoform-b; O, OCT4; K, KLF4; HOK, primary Human Oral Keratinocyte; HCEC, human corneal endothelial cells; HUVEC, normal human umbilical vein endothelial cells; NHDF-Ad, adult normal human dermal fibroblasts; iPSC, induced pluripotent stem cell. The data are presented as the mean ± SEM (**a**,**b**).

We next examined the effect of the epigenetic state on KRT12 induction, for which a reprogramming factor, OCT4, is required. The expression of *NANOG*, a pluripotency marker regulated by OCT4^26^,^27^, was slightly elevated in *KRT12*-expressing OKF6/TERT-1 cells (Fig. 6a). Overall, however, the pluripotency markers, including NANOG, SSEA4, and TRA-1-60, were not strongly detected by immunofluorescence staining, suggesting that their expression levels are not as high as in induced pluripotent stem cells (iPSCs), which are in an undifferentiated state (Supplementary Fig. S5a–c). *KDR*, a marker of early mesodermal cells, and *SOX17*, a marker of early endodermal cells, were both slightly up-regulated in *KRT12*-positive cells under certain conditions (Fig. 6a), suggesting that the epigenetic state modified by OCT4 is one of the possible factors that affect the expression of *KRT12*^28^,^29^.

We added small molecules that are known to contribute to the maintenance of the undifferentiated state and the improvement of the efficiency of reprogramming to iPSCs (Fig. 6b)^30^,^31^,^32^. The induction of *KRT12* and *KRT3* following PAX6-b-OCT4-KLF4 transduction was enhanced by addition of BIX01294 (BIX), a selective inhibitor of G9a histone methyltransferase. This result also suggests the involvement of a modified epigenetic state in the induction of *KRT12* and *KRT3* by PAX6-b-OCT4-KLF4.

Consistent with the high expression of *Wnt2* in the mouse embryonic corneal epithelium (Supplementary Table S1), the induction of *KRT12* and *KRT3* was enhanced by 6-bromoindirubin-3′-oxime (BIO), a glycogen synthase kinase-3 (GSK-3) inhibitor (Fig. 6b), which supports the involvement of Wnt signalling in the corneal epithelial phenotype^33^,^34^.

### Corneal epithelium-specific keratins are preferentially induced in surface ectoderm-derived cells

Next, we transduced *PAX6-a*, *PAX6-b*, *OCT4,* and *KLF4* into different types of human cells (Fig. 6c and Supplementary Fig. S5d). All surface ectoderm-derived cells examined (OKF6/TERT-2 cells from another oral mucosal epithelial cell line, N/TERT-1 and N/TERT-2 cells from dermal epithelial cell lines, and human oral keratinocytes [HOK] from a primary oral epithelium) expressed *KRT12* at high levels after being transduced with PAX6-b-OCT4-KLF4. The same cells expressed *KRT3* at high levels after being transduced with PAX6-a-OCT4-KLF4. Slightly lower expression levels of *KRT3* were induced by PAX6-a in the absence of OCT4 and KLF4. The non-surface ectoderm-derived epithelial cells (ARPE-19, MKN1, and HepG2) tended to express *KRT12* and *KRT3* mRNA, but few KRT12- and KRT3-positive cells were detected by immunofluorescence staining. iPSCs that express OCT4 endogenously did not show any *KRT12* and *KRT3* expression following the transduction of PAX6 and KLF4, suggesting that *KRT12* and *KRT3* induction in the surface ectoderm-derived cells did not progress to a de-differentiated state.

## Discussion

Limbal stem cell deficiency, which is very difficult to treat, is one of the most severe diseases of the ocular surface and causes a significant corneal epithelial defect and vision impairment^35^. Several research groups, including ours, have attempted to successfully transplant cultured autologous oral mucosal epithelial sheets to treat eyes with bilateral limbal stem cell deficiencies^12^,^13^. This approach has shown some promise; however, the reconstructed ocular surface does not allow a full, long-term improvement of visual acuity, and is prone to problems associated with neovascularization. This is probably because at least in part the oral mucosal epithelium lacks corneal epithelial specific genes, such as PAX6 and KRT12^9^,^10^. To minimize such limitations, a much greater understanding of the molecular mechanisms underlying the regulation of corneal epithelium-specific genes, especially the genes controlled by PAX6, is urgently required. Here, we report, for the first time, the effect of two isoforms of PAX6 on corneal epithelium-specific genes, particularly *KRT3* and *KRT12*.

PAX6-a and PAX6-b display differential inductive properties on *KRT3* and *KRT12* and regulate different genes by forming a highly complex regulatory network. Thus, these two isoforms of PAX6 cooperatively regulate genes in the epithelium. Moreover, the upstream region of the *KRT3* gene appears to be a direct target of PAX6-a transduction. Although previous work has concluded that PAX6-a binds to its target by PAI domain and PAX6-b binds to its target by RED domain^3^,^5^, we show that the entire PAI and RED domains are critical for *KRT3* and *KRT12* induction.

Following the addition of the Yamanaka factors, we discovered that KLF4 was important for the efficient induction of *KRT3* and *KRT12*. At the outset of these experiments, we expected that KLF4 would work as a reprogramming factor^17^,^18^,^36^, but it appeared to act as an accelerator of the expression of differentiation marker keratins^37^,^38^. Additionally, a modified epigenetic state by OCT4 and BIX may be one of the interventions that improves *KRT12* induction in oral mucosal epithelial cells^29^,^39^. The low expression levels of OCT4 in the corneal epithelium also point to the fact that it likely works indirectly on the regulatory region of *KRT12*, through the modification of its epigenetic state in oral mucosal epithelial cells. However, unlike *KRT12* expression, sufficient *KRT3* expression was induced without OCT4 (i.e., with PAX6-a or PAX6-a-KLF4), suggesting that the *KRT3* regulatory region is readily accessible for the binding of transcription factors without epigenetic modification.

The efficiency of *KRT12* induction was found to be relatively low, even though we used a uniform cell line. This can be explained by the requirement for high expression levels of the transgenes for *KRT12* induction, which provides new insight into the low efficiency of iPSC generation by transduction with the four Yamanaka factors^17^,^18^,^40^. As most cells expressed *KRT3* following PAX6-a-OCT4-KLF4 induction, the high expression of the transgenes does not seem to be necessary for the induction of *KRT3*. Our experiments revealed that the cell source was another important factor for the induction of tissue-specific genes, even when utilizing epigenetic modifications by OCT4. As summarized by Chin, most studies relating to direct reprogramming used fibroblasts in their experiments^28^. Our data suggest that the selection of appropriate cell types makes it easier to overcome the inherent epigenetic differences among tissue-specific genes and increases the chance of achieving direct reprogramming. In fact, PAX6 is able to induce *KRT12* expression on its own in epithelial cells from the corneal pannus or eyelid, the origins of which are close to those of the corneal epithelium^34^,^41^.

**Figure 7:**
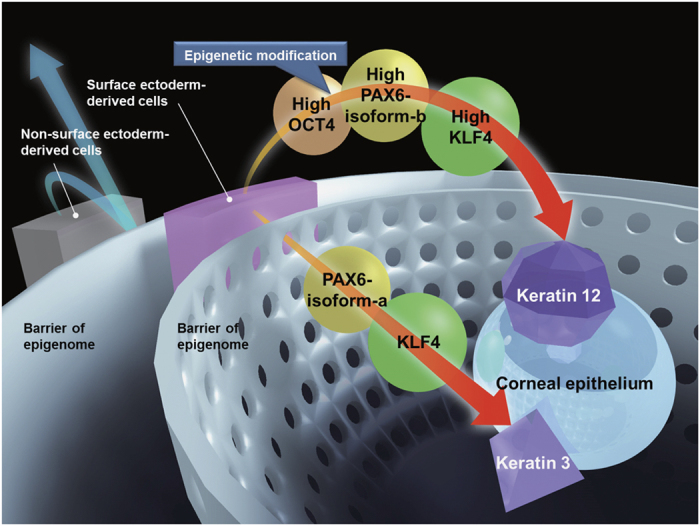
A schematic representation of the proposed pathways. The epigenetic differences between the cell lineages are depicted as barriers of the epigenome. The roles of each transcription factor in the induction of corneal epithelium-specific genes are as follows: (1) PAX6-a and PAX6-b transduction induces KRT3 and KRT12 expression, respectively, (2) KLF4 accelerates the differentiation, (3) highly expressed transgenes are required for KRT12 induction, (4) epigenetic modifications brought about by OCT4 are required to induce KRT12 expression. Unlike KRT12 induction, KRT3 induction did not require OCT4 because the epigenetic state of *KRT3* in the surface ectoderm-derived cells was already accessible for the binding of transcription factors. As a cell source, the surface ectoderm-derived cells were able to effectively induce KRT3 and KRT12 expression.

In summary, we report that two PAX6 isoforms, alongside OCT4 and KLF4, differentially and cooperatively regulate corneal epithelium-specific genes, particularly *KRT3* and *KRT12*, as well as many other genes in surface ectoderm-derived cells. The role of each transcription factor is summarized in Fig. 7. Our new findings will contribute to further our understanding of the molecular basis of the corneal epithelium specific phenotype.

## Methods

### Laser micro-dissection of human corneal epithelium and mouse embryos *in vivo*

Monolayers of the corneal epithelium, along with the basal layer of the oral mucosal epithelium, were micro-dissected from frozen sections of ICR mouse embryos (Japan SLC, Hamamatsu, Japan) using a PALM MicroBeam (Carl Zeiss Microscopy GmbH, Göttingen, Germany). Frozen sections of the human corneal epithelium (SightLife, Seattle, WA, USA) were micro-dissected *in vivo* from four areas; the central-apical, central-basal, limbal-apical and limbal-basal cornea areas. The conjunctival epithelium was obtained by similar micro-dissections.

### Cell culture

Human corneal epithelial cells were cultured in Dulbecco’s Modified Eagle’s Medium (DMEM): F12 Medium (1:1) (Life Technologies, Carlsbad, CA, USA) supplemented with a B-27^®^ supplement (Life Technologies)` 10 μM of Y-27632 (Wako Pure Chemical Industries, Osaka, Japan), 20 ng/ml of human recombinant KGF/FGF-7 (R & D Systems, Minneapolis, MN, USA) and 2 mM of L-Glutamine (Life Technologies)^42^, and incubated in 5% CO_2_ at 37 °C. Immortalized human keratinocytes (OKF6/TERT-1, OKF6/TERT-2, N/TERT-1, N/TERT-2 cells) were obtained from the laboratory of Dr. J. Rheinwald (Harvard Institutes of Medicine, Boston, MA, USA)^19^. Cells were cultured in Keratinocyte-SFM supplemented with 25 μg/ml of bovine pituitary extract (BPE), 0.2 ng/ml of epidermal growth factors (EGF, Life Technologies) and 0.3 mM of CaCl_2_ (Wako Pure Chemical Industries). Again, the cells were incubated in 5% CO_2_ at 37 °C. The other human cells, which were used in the current study and their culture media are listed in Supplementary Table S6.

### Viral transduction

Two isoforms of PAX6, along with their DNA-binding domain truncated mutants (PAX6ΔPAI, PAX6-aΔRED and PAX6-bΔRED), OCT4, KLF4 and *lacZ* (control) were subcloned into a pLenti7.3/V5-DEST^TM^ Vector (Life Technologies). The Yamanaka factors (OCT4, SOX2, KLF4 and c-Myc), which had been subcloned into CSV-CMV-MCS-IRES2-Venus plasmids, were kindly provided by Dr. Hiroyuki Miyoshi, from the RIKEN BioResource Center. In addition, we constructed all-in-one vectors, which express ‘PAX6 and KLF4’ or ‘PAX6, OCT4 and KLF*4*’ at the same time on pLenti7.3/V5-DEST^TM^ Vectors, by using the *2A* sequence^43^. The lentiviruses were produced using the ViraPower^TM^ Lentiviral Expression System (Life Technologies). The lentiviral vectors provided by Dr. Miyoshi were co-transfected into 293T cells with pCMV-VZV-G-RSV-Rev, pCAG-HIV-gp (again, kindly provided by Dr. Miyoshi) and FuGene^®^ HD (Roche Applied Science, Manheim, Germany). The collected virus-containing supernatants were ultra-centrifuged in an Optima L-90K Preparative Ultracentrifuge (Beckman Coulter, Brea, CA, USA) for 1.5 h at 50000 × g. After the viruses and 6 μg/ml of polybrene (Nacalai tesque, Kyoto, Japan) were added to the cells, they were cultured for 24 h followed by an additional incubation for 48 h in fresh medium.

### Immunofluorescence staining

The fixed samples were blocked and permeabilized with buffer containing 5% normal donkey serum (Jackson ImmunoResearch Laboratories, West Grove, PA, USA) and 0.3% Triton X-100 (Sigma-Aldrich, St. Louis, MO, USA), and incubated with various antibodies, as described in the Supplementary Information.

### Quantitative reverse transcription PCR (qRT-PCR)

The total RNA was extracted from the cells, and the synthesized cDNA underwent quantitative PCR amplification with TaqMan Gene Expression Assay probes (Supplementary Table S7) and a Taqman^®^ Fast Universal PCR Master Mix (Life Technologies). We also designed two types of PAX6 primers and probes, to distinguish the expression of the two variants of *PAX6,* as described in the Supplementary Information. For the SYBR green quantitative PCR, cDNA was applied to quantitative PCR amplification with designed primers (Supplementary Table S8) and SYBR Premix Ex Taq^TM^ GC (TaKaRa Bio, Otsu, Japan). cDNAs from the islet cells (Primary Cell Co., Sapporo, Japan) and ocular tissue (SightLife) were used as the positive controls for the qRT-PCR. The final mRNA levels were normalized to the *GAPDH* levels.

### Single-cell gene expression analysis

The single-cell gene expression analysis was performed using a Fluidigm Single-Cell Gene Expression Workflow system (Fluidigm, San Francisco, CA, USA). The Taqman probes used are listed in Supplementary Table S7.

### Preparation of RNA-seq libraries and analysis of RNA-seq data

RNA libraries from four replicates of each transduced cell type were assembled using ISOGEN (Wako Pure Chemical Industries) and the TruSeq RNA Sample Prep kit, v2 (Illumina, San Diego, CA, USA), according to the manufacturer’s protocol. After purification and fragmentation, the mRNAs were sequenced by Illumina HiSeq 2000/2500, which generated 20.9–81.8 million 101-bp paired-end reads. The sequenced reads were mapped and quantified by the TopHat2 (v.2.0.7)/Cufflinks (v.2.0.5) pipeline^44^, with 84–91% of total reads uniquely mapped (Supplementary Table S9) and that normalized as the unit FPKM (fragments per kilobase of exon per million mapped reads). Differentially expressed genes were detected by Cuffdiff program in the Cufflinks package with parameters set as <5% false discovery rate (FDR) and >2.0 fold change (FC), as described in the Supplementary Information.

### Regression promoter modelling

The procedure of computational promoter modelling^24^ was performed to detect the potential key regulators (Supplementary Information). This approach exhaustively searches well-fitted linear regression models that infer the importance of TFBSs to explain the gene expression levels. The TFBSs and the relevant transcription factors were prepared with the TRANSFAC professional^23^ and MATCH tools^45^. The statistical significance of the inferred TFBSs was tested by a one-sample t-test after a Bonferroni correction (<0.01 *p*-value). The networks (Supplementary Fig. S3b,e and Fig. 4e) were visualized using Cytoscape software (www.cytoscape.org).

### Dual secreted reporter assay

The dual secreted reporter assay was performed using a Ready-To-Glow^TM^ Dual Secreted Reporter Assay (Clontech Laboratories, Mountain View, CA, USA). We subcloned various lengths of the DNA upstream of *KRT12* and *KRT3* into pMetLuc2-Reporters, which were termed *KRT12-1K*, *KRT12-2K*, *KRT12-3K*, *KRT12-4K*, *KRT12-5K*, *KRT12-6K*, *KRT3-1K*, *KRT3-2K*, *KRT3-3K*, *KRT3-4K*, *KRT3-5K* and *KRT3-6K* reporters, respectively. Three types of vectors, i.e. a pSEAP2-control, one of the pMetLuc2-reporters and one of the pLenti7.3/V5-DEST^TM^ vectors, were co-transfected using Lipofectamine^®^ 3000 (Life Technologies). The culture medium was collected 24 h following the transfection, after which a SEAP assay and a secreted metridia luciferase assay were performed.

### Co-immunoprecipitation (Co-IP) and mass spectrometry (MS)

We used an EpiXplore^TM^ Nuclear Co-Immunoprecipitation Kit (Clontech Laboratories) to identify the protein-protein complexes. The nuclear extracts were incubated with a rabbit anti-PAX6 antibody (1:50, Abcam, Cambridge, UK) for 24 h. The purified proteins were subjected to sodium dodecyl sulfate poly-acrylamide gel electrophoresis (SDS-PAGE), followed by the extraction of the target lanes and an analysis using liquid chromatography-mass spectrometry (LC-MS/MS, Thermo Fisher Scientific, Waltham, MA, USA).

### Tetracycline-on (Tet-On) system

We generated a ViraPower^TM^ T-Rex^TM^ OKF6/TERT-1 cell line through the transduction of pLenti3.3/TR (Life Technologies). The all-in-one cassettes were subcloned into a pLenti6.3/TO/V5-DEST vector (Life Technologies). After we transduced a ViraPower^TM^ T-Rex^TM^ OKF6/TERT-1 cell line with pLenti6.3/TO/V5-DEST vectors, we selected four PAX6-a/OCT4/KLF4- and PAX6-b/OCT4/KLF4-inducible colonies.

### Treatment with small molecules

The small molecules used in these experiments were 600 nM of 6-Bromoindirubin-3′-oxime (BIO, Wako Pure Chemical Industries), 3 μM of BIX01294 (BIX, Stemgent, San Diego, CA, USA), 120 nM of RG108 (Stemgent), 6 μM of R(+)BayK 8644 (BayK, Stemgent) and 300 μM of Valproic Acid (VPA, Stemgent).

### Bioinformatics analysis

The enrichment analysis of Gene Ontology (GO) biological process terms was performed with the GOFunction package of Bioconductor (v.3.0, http://www.bioconductor.org/) with a Bonferroni *p*-value correction (<0.01). The R programming language (http://www.r-project.org/) was used for the regression modelling. The statistical significance of the set of 1000 RCs was tested by a one-sample t-test after Bonferroni correction (<0.01 *p*-value). The other statistical analyses were performed with a t-test, a two-sided Dunnett’s test and a paired t-test with Bonferroni correction. A correlation coefficient (r) was applied to the correlation between two genes, and *p* < 0.05 was considered statistically significant.

## Additional Information

**Data availability:** The RNA-seq data generated in this study have been deposited in the DDBJ (DNA Data Bank of Japan) Sequence Read Archive (DRA), under the accession number DRA002960.

**How to cite this article**: Sasamoto, Y. *et al.* PAX6 Isoforms, along with Reprogramming Factors, Differentially Regulate the Induction of Cornea-specific Genes. *Sci. Rep.* **6**, 20807; doi: 10.1038/srep20807 (2016).

## Supplementary Material

Supplementary Information

Supplementary Tables S1-S4, S9

Supplementary Table 5
