# Supplementary material for: PAX6 Isoforms, along with Reprogramming Factors, Differentially Regulate the Induction of Cornea-specific Genes
Source: Sci Rep. 2016 Feb 22;6:20807. doi: 10.1038/srep20807 (PMC4761963; doi:10.1038/srep20807)
Supplement: Supplementary Information [file srep20807-s1.pdf]

Scientific Reports

Supplementary Information

PAX6 isoforms, along with reprogramming factors, differentially regulate the induction of cornea-specific genes

Yuzuru Sasamoto, Ryuhei Hayashi, Sung-Joon Park, Mihoko Saito-Adachi, Yutaka Suzuki, Satoshi Kawasaki, Andrew J. Quantock, Kenta Nakai, Motokazu Tsujikawa, Kohji Nishida

Supplementary Figure S1

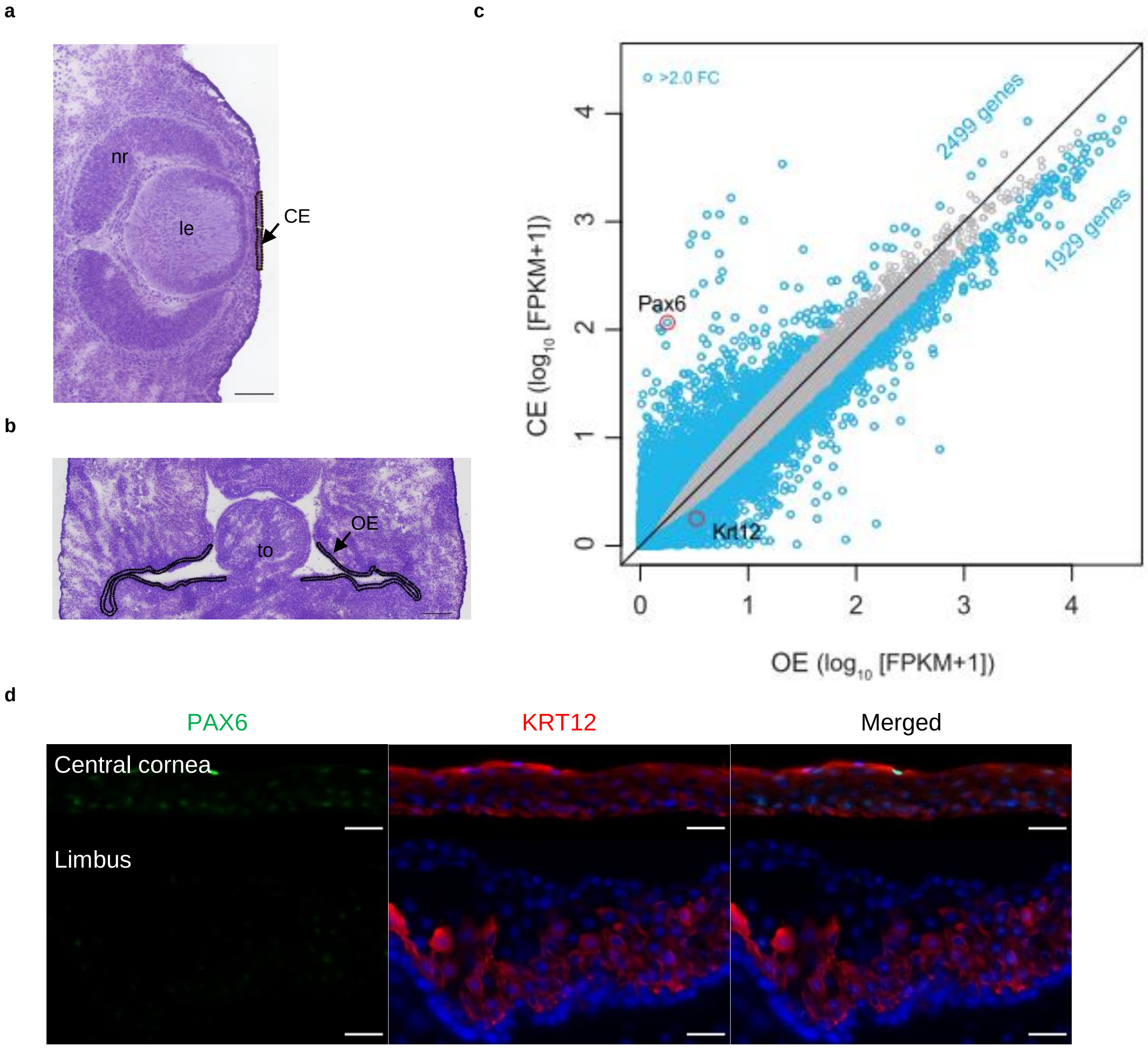

Supplementary Figure S1. Gene expression of markers in the corneal epithelium.

(**a**, **b**) Micro-dissection of corneal epithelial cells (**a**) and oral mucosal epithelial cells (**b**) of ICR mice at E12.5. The dotted lines indicate where the micro-dissections were performed.

(**c**) Correlation between the expression of oral epithelium- and corneal epithelium-dependent differentially up-regulated genes (DUGs) in E12.5 mice.

(**d**) Immunofluorescence staining of PAX6 and KRT12 in human corneal epithelium *in vivo*.

The scale bars represent 100 μm (**a**), 200 μm (**b**), or 25 μm (**d**).

CE, corneal epithelium; OE, oral epithelium; nr, neural retina; le, lens; to, tongue; FC, fold change; FPKM, fragments per kilobase of exon per million mapped reads.

Supplementary Figure S2

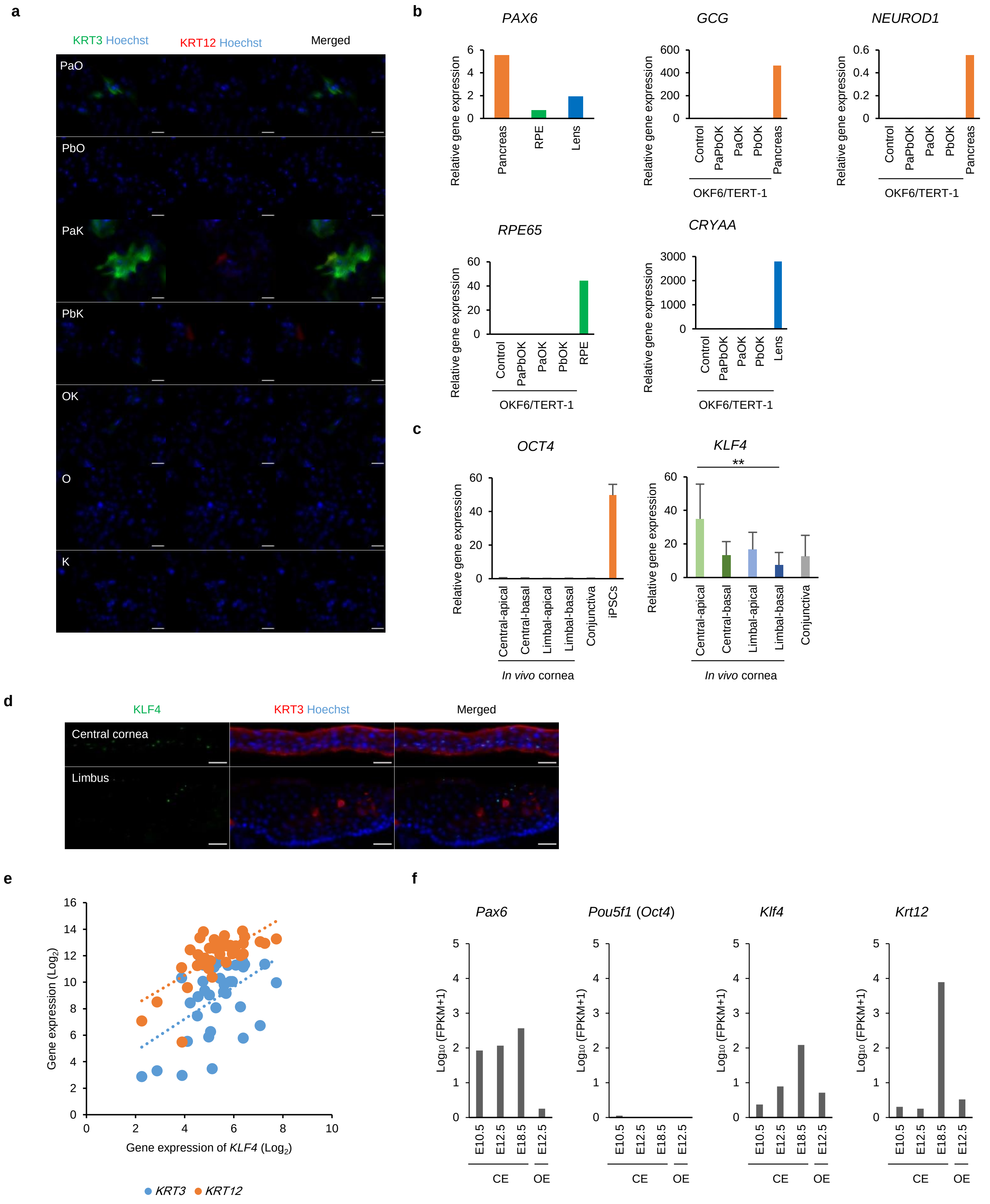

Supplementary Figure S2. Gene expression of OKF6/TERT-1 cells following various patterns of transduction and the expression of transcription factors in the corneal epithelium.

(**a**) Immunofluorescence staining of KRT3 and KRT12 following various patterns of transduction.

(**b**) qRT-PCR of other marker genes of *PAX6*-positive tissues at day 3 (n = 4 for OKF6/TERT-1 cells and n = 1 for positive control). *GCG* for pancreas islet cells, *NEUROD1* for pancreas islet cells and neurons, *RPE65* for RPE, *CRYAA* for the lens epithelium. The data are presented as the mean ± SEM.

(**c**) qRT-PCR analysis of *OCT4* and *KLF4* mRNA levels in four areas of the corneal epithelium *in vivo* (central-apical, central-basal, limbal-apical and limbal-basal) and conjunctival epithelium *in vivo* (n = 4). The feeder-free iPSCs is a positive control for *OCT4* mRNA levels (n = 4). The data are presented as the mean ± standard deviation (SD). **$p < 0.05$ versus central-apical corneal epithelium by Dunnett's test.

(**d**) Immunofluorescence staining of KLF4 and KRT3 in human corneal epithelium *in vivo*.

(**e**) Correlation between gene expression levels of *KLF4* and *KRT3*, or *KRT12*, in limbal epithelial cells *in vivo* (n = 37), assessed by single-cell gene expression analysis (r = 0.51, $p < 0.01$ and r = 0.69, $p < 0.01$, respectively).

(**f**) mRNA expression levels of *Pax6*, *Oct4*, *Klf4*, and *Krt12* in mouse embryonic corneal epithelium (E10.5, E12.5 and E18.5) and mouse embryonic oral epithelium (E12.5) by RNA-seq using the micro-dissected samples.

Pa, PAX6-isoform-a; Pb, PAX6-isoform-b; O, OCT4; K, KLF4; RPE, retinal pigment epithelium; iPSCs, induced pluripotent stem cells; *Krt12*, murine *keratin 12*; FPKM, fragments per kilobase of exon per million mapped reads; CE, corneal epithelium; OE, oral epithelium. The scale bars represent 50 μm (**a**) or 25 μm (**d**).

Supplementary Figure S3

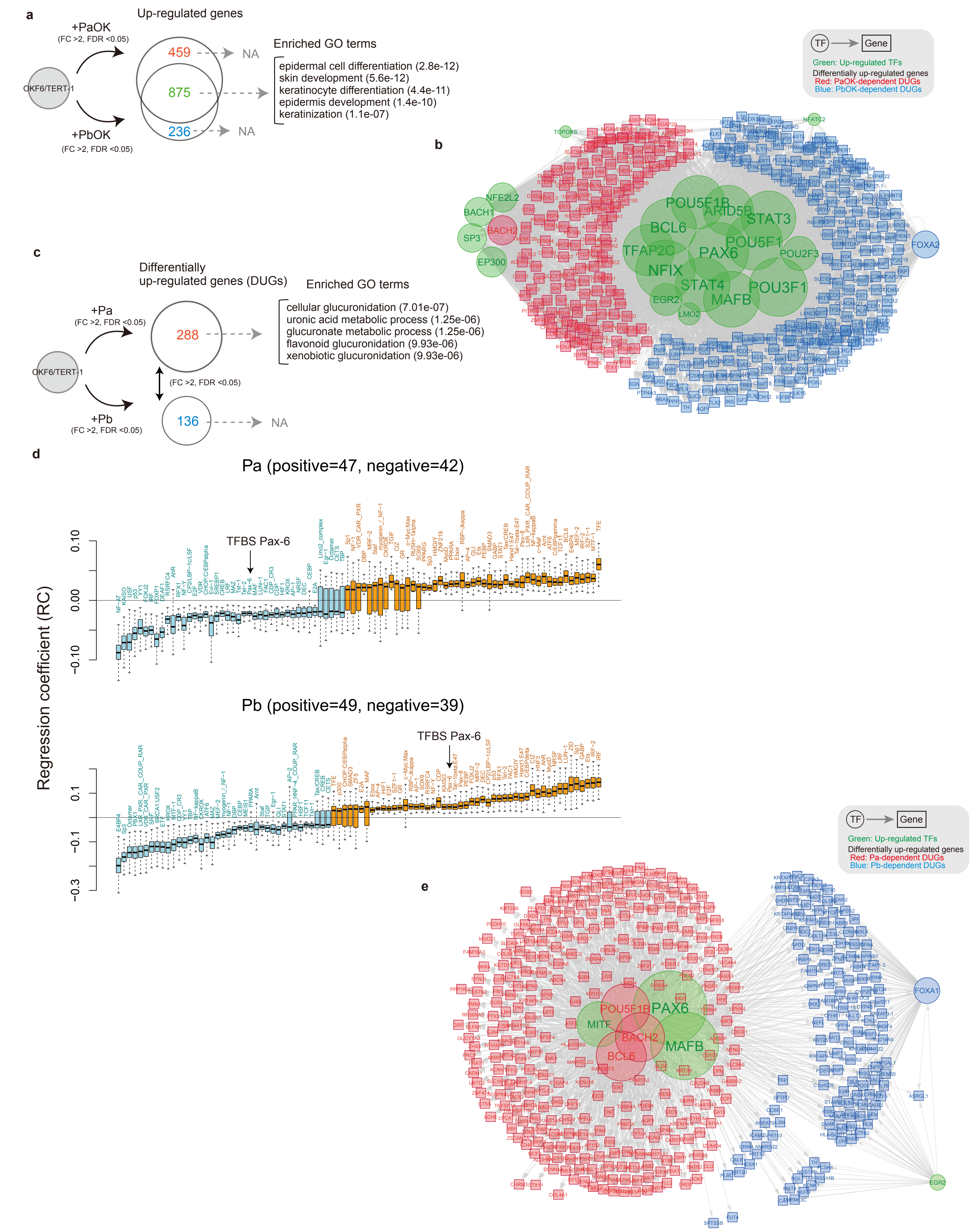

Supplementary Figure S3. Transcriptome analysis and inference of the regulatory network in OKF6/TERT-1 cells.

(**a**) Schematic representation of the identification of up-regulated genes in PAX6-a-OCT4-KLF4- and PAX6-b-OCT4-KLF4-transduced cells.

(**b**) Gene regulatory network inferred from PAX6-a-OCT4-KLF4 and PAX6-b-OCT4-KLF4 transductions.

(**c**) Schematic representation of the identification of differentially up-regulated genes (DUGs) following PAX6-a and PAX6-b transductions.

(**d**) Distribution of the regression coefficients for 99 putative TFBSs to explain the expression levels of PAX6-a- (upper panel) and PAX6-b-dependent DUGs (lower panel). The data are presented as quantile plots. TFBS Pax-6 is a putative binding site for PAX6 isoforms. On average, $r^2 = 0.85$ for PAX6-a-dependent DUGs and $r^2 = 0.96$ for PAX6-b-dependent DUGs.

(**e**) Gene regulatory network inferred from PAX6-a and PAX6-b transductions.

Pa, PAX6-isoform-a; Pb, PAX6-isoform-b; O, OCT4; K, KLF4; FC, fold change; FDR, false discovery rate; NA, not available; GO, gene ontology; TF, transcription factor; DUG, differentially up-regulated gene; TFBS, TF binding site. The numbers in the parentheses represent the *p*-values of the hypergeometric test with a Bonferroni correction (**a**, **c**).

Supplementary Figure S4

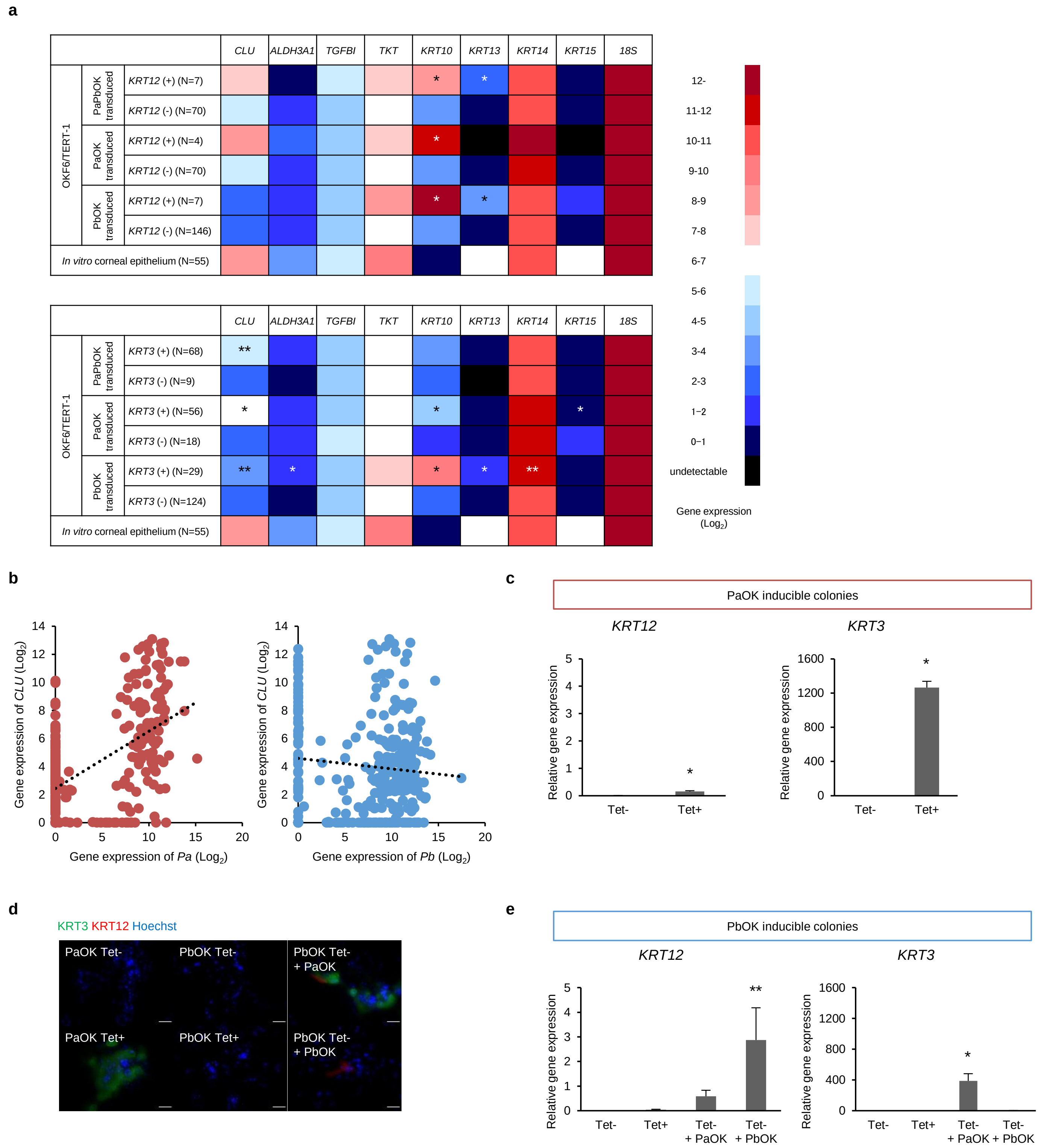

Supplementary Figure S4. Single-cell gene expression analysis of transduced OKF6/TERT-1 cells and KRT12 and KRT3 induction with all-in-one vectors.

(**a**) Gene expression patterns of PAX6-a-PAX6-b-OCT4-KLF4-, PAX6-a-OCT4-KLF4- and PAX6-b-OCT4-KLF4-transduced OKF6/TERT-1 cells at the single-cell level. All cells were sub-grouped by expression level of *KRT12* (upper) or *KRT3* (lower). The scale numbers are the $\log_2$ of gene expression. *$p < 0.01$ and **$p < 0.05$ by t-test.

(**b**) Correlations between gene expression levels of *PAX6-a* and *CLU* (left) and *PAX6-b* and *CLU* (right) at the single-cell level. *CLU* expression was positively correlated with that of *PAX6-a* ($r = 0.568$, $p < 0.01$), but not with that of *PAX6-b* ($r = -0.101$, $p = 0.08$).

(**c**) Expression of *KRT12* and *KRT3* mRNA levels of PAX6-a-OCT4-KLF4-inducible colonies without (Tet−) and with (Tet+) tetracycline (n = 4). *$p < 0.01$ versus Tet− state obtained with a t-test.

(**d**) Expression of KRT12 and KRT3 determined by immunofluorescence staining in Tet- and Tet+ states. PAX6-b-OCT4-KLF4-inducible colonies in the absence of tetracycline (Tet−) were also transduced with PAX6-a-OCT4-KLF4 or PAX6-b-OCT4-KLF4. Each scale bar represents 50 μm.

(**e**) Expression levels of *KRT12* and *KRT3* mRNA in PAX6-b-OCT4-KLF4-inducible colonies in Tet− and Tet+ states (n = 4). The PAX6-b-OCT4-KLF4-inducible colonies in the absence

of tetracycline (Tet−) were also transduced with PAX6-a-OCT4-KLF4 or PAX6-b-OCT4-KLF4.

*$p < 0.01$ and **$p < 0.05$ versus Tet− state determined with a Dunnett's test.

Pa, PAX6-isoform-a; Pb, PAX6-isoform-b; O, OCT4; K, KLF4. The data are presented as the mean ± SEM (**c**, **e**).

Supplementary Figure S5

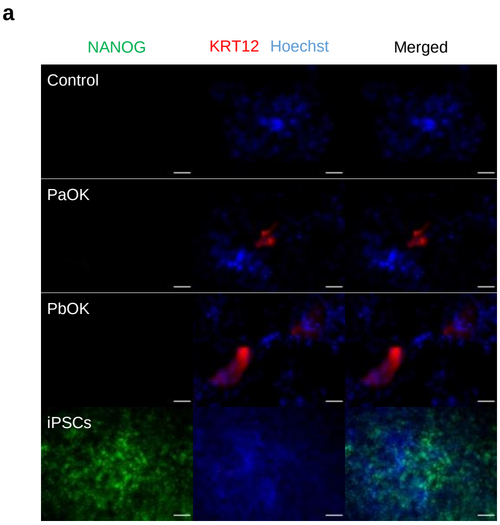

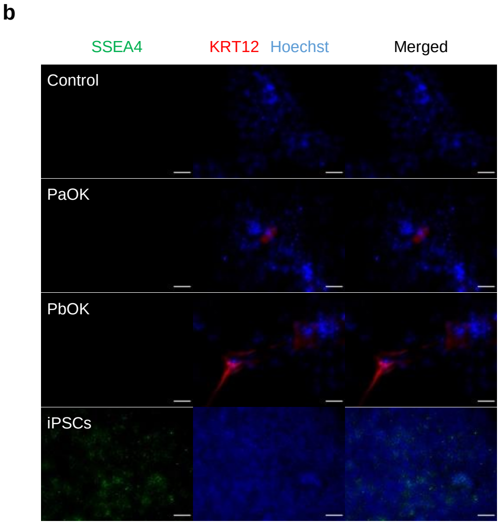

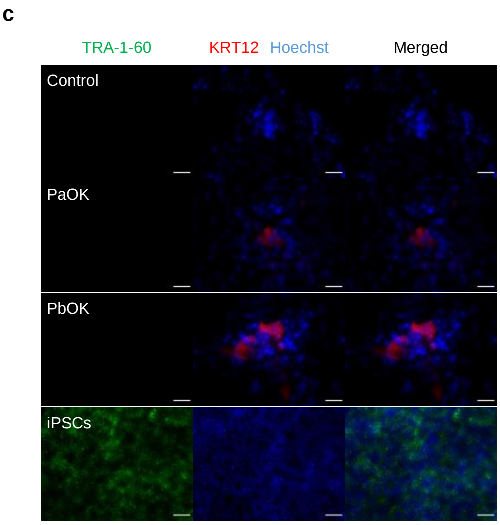

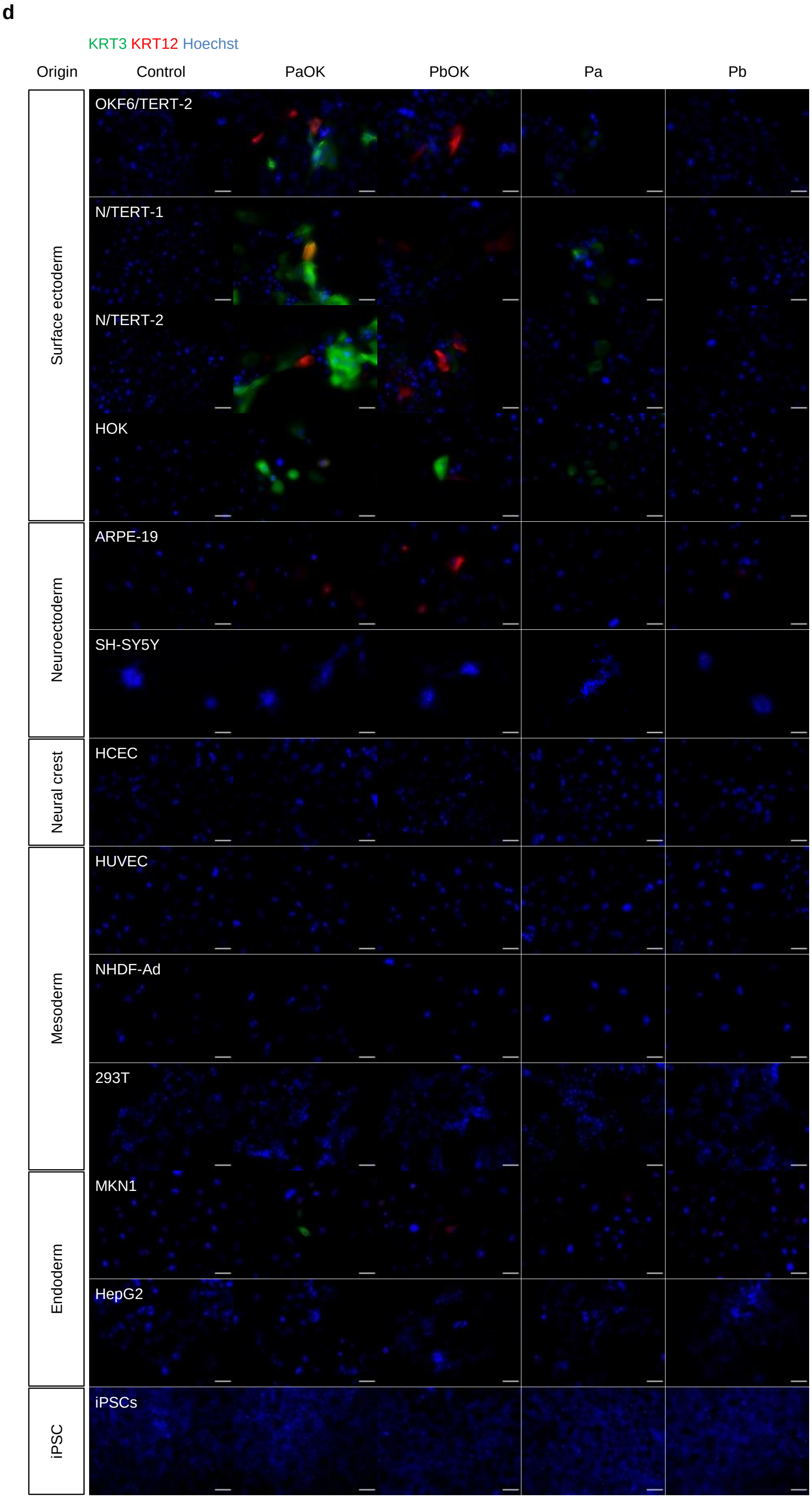

Supplementary Figure S5. Effect of transgenes on epigenetics and different cells.

(**a**-**c**) Immunofluorescence staining of (**a**) NANOG, (**b**) SSEA4 and (**c**) TRA-1-60 and KRT12 in transduced OKF6/TERT-1 cells at day 3. iPSCs were used as positive controls.

(**d**) Immunofluorescence staining of KRT12 and KRT3 in transduced cells.

Pa, PAX6-isoform-a; Pb, PAX6-isoform-b; O, OCT4; K, KLF4; iPSCs, induced pluripotent stem cells; HOK, primary Human Oral Keratinocyte; HCEC, human corneal endothelial cells; HUVEC, normal human umbilical vein endothelial cells; NHDF-Ad, adult normal human dermal fibroblasts. Each scale bars represents 50 μm.

Supplementary Tables

Supplementary Table S1. Differentially expressed genes in the corneal and oral mucosal epithelia (ICR mice at E12.5).

See the corresponding Excel file. CE, corneal epithelium; OE, oral epithelium; FC, fold change.

Supplementary Table S2. PAX6-binding proteins detected by co-immunoprecipitation, followed by mass spectrometry.

See the corresponding Excel file. Pa, PAX6-isoform-a; Pb, PAX6-isoform-b; O, OCT4; K, KLF4.

Supplementary Table S3. Differentially up-regulated genes by (1) PAX6-a-OCT4-KLF4 transduction (162 genes) and (2) PAX6-b-OCT4-KLF4 transduction (220 genes).

See the corresponding Excel file. Pa, PAX6-isoform-a; Pb, PAX6-isoform-b; O, OCT4; K, KLF4.

Supplementary Table S4. Regression coefficients of 103 TFBSs identified by exhaustive regression modelling.

See the corresponding Excel file. TFBS, transcription factor binding site; Pa, PAX6-isoform-a; Pb, PAX6-isoform-b; O, OCT4; K, KLF4.

Supplementary Table S5. Network of Supplementary Fig. S3b, e and Fig. 4e.

See the corresponding Cytoscape session files. Pa, PAX6-isoform-a; Pb, PAX6-isoform-b; O, OCT4; K, KLF4.

Supplementary Table S6. Human cells used in the present study and their respective culture medium.

Human cells	Culture medium
Human embryonic kidney cell line 293T cells (293T, RIKEN BioResource Center Cell Bank, Tsukuba, Japan)	Dulbecco's Modified Eagle's Medium (DMEM, Life Technologies, Carlsbad, CA, USA) supplemented with 10 % foetal bovine serum (FBS, Japan Bio Serum, Fukuyama, Japan).
293FT cells (Life Technologies)	DMEM supplemented with 10 % FBS, 0.1 mM of MEM Non-Essential Amino Acids (NEAA, Life Technologies) and 6 mM of L-Glutamine (Life Technologies)
Primary Human Oral Keratinocyte	CnT-24 (CELLnTEC Advanced Cell Systems AG,

(HOK, ScienCell Research Laboratories, Carlsbad, CA, USA)	Bern, Switzerland)
ARPE-19 (ATCC, Manassas, VA, USA)	DMEM: F12 Medium (ATCC) supplemented with 10 % FBS
SH-SY5Y (ATCC)	1:1 of Eagle's Minimum Essential Medium (ATCC) and Ham's F-12 Nutrient Mix (F-12, Life Technologies) supplemented with 10 % FBS
Human corneal endothelial cells (HCEC-B4G12, Creative Bioarray, Shirley, NY, USA)	Human Endothelial-SFM (Life Technologies) supplemented with 10 ng/mL of human recombinant basic fibroblast growth factor (bFGF, Wako Pure Chemical Industries, Osaka, Japan) in 10 cm of culture dishes coated with 5 mg of chondroitin-6-sulfate (Sigma-Aldrich, St. Louis, MO, USA) and 10 μg of laminin (Sigma-Aldrich)
Normal human umbilical vein endothelial cells (HUVEC, Cell Systems, Troisdorf, Germany)	EBM Basal Medium (Lonza, Basel, Switzerland) supplemented with EGM® SingleQuot Kit Suppl. & Growth Factors (Lonza)
Adult Normal Human Dermal Fibroblasts	DMEM (Life Technologies) supplemented with 10 %

(NHDF-Ad, Lonza)	FBS
MKN1 (RIKEN BioResource Center, Tsukuba, Japan)	RPMI 1640 Medium (Life Technologies) supplemented with 10 % FBS
HepG2 (RIKEN BioResource Center)	Minimum Essential Medium (MEM, Life Technologies) supplemented with 10 % FBS and 0.1 mM of NEAA
Adult HDF-derived human induced pluripotent stem cell (hiPSC) line 201B7 (RIKEN BioResource Center)[17]	1) Mouse embryonic fibroblast (MEF, KITAYAMA LABES Co. LTD., Nagano, Japan) based iPS culture; ES culture medium containing DMEM/F12 (Life Technologies) supplemented with 20 % Knockout™ Serum Replacement (KSR, Life Technologies), 0.1 mM of 2-Mercaptoethanol (2-ME, Life Technologies), 0.1 of mM NEAA, and 4 ng/mL of bFGF. MEF was treated with Mitomycin-C (Kyowa Hakko Kirin, Tokyo,, Japan). 2) BD Matrigel™ Basement Membrane Matrix (BD Biosciences, Franklin Lakes, NJ, USA) coated feeder-free culture; MEF-conditioned ES culture medium.

MKN1 and HepG2 were provided by the RIKEN BioResource Center through the National Bio-Resource Project of the MEXT, Japan.

Supplementary Table S7. Primers and probes for the Taqman® gene expression assay.

Gene	Taqman probe
GAPDH	Hs99999905_m1
PAX6	Hs00240871_m1
OCT4	Hs00999632_g1
KLF4	Hs00358836_m1
KRT3	Hs00365080_m1
KRT10	Hs00166289_m1
KRT12	Hs00165015_m1
KRT13	Hs00999762_m1
KRT14	Hs00559328_m1
KRT15	Hs00267035_m1
KRT19	Hs00761767_s1
KRT76	Hs01052531_g1
CLU	Hs00971656_m1
ALDH3A1	Hs00964880_m1
TGFBI	Hs00932732_m1

TKT	Hs01115545_m1
GCG	Hs01031536_m1
NeuroD	Hs0192295_s1
RPE65	Hs01071462_m1
CRYAA	Hs00166138_m1
NANOG	Hs02387400_g1
KDR	Hs00911700_m1
SOX17	Hs00751752_s1
18S	Hs99999901_s1

Supplementary Table S8. Primers for the SYBR green gene expression assay.

	Forward Primer	Reverse Primer
GAPDH	5'-AGCCTCCCGCTTCGCTCTCT-3'	5'-CCAGGCGCCCAATACGACCA-3'
PAX6-a	5'-CGAGATTTCAGAGCCCCATA-3'	5'-CGTTGGACACCTGCAGAAT-3'
PAX6-b	5'-CGAGATTTCAGAGCCCCATA-3'	5'-TTTGATTGTCCAGCACTTGG-3'

Supplementary Table S9. Summary of mapping rates of paired R1 and R2 reads.

See the corresponding Excel file. Pa, PAX6-isoform-a; Pb, PAX6-isoform-b; O, OCT4; K, KLF4, CE, corneal epithelium; OE, oral epithelium.

Supplementary Methods

Laser micro-dissection of the mouse embryos and the human corneal epithelium

ICR mice (Japan SLC, Hamamatsu, Japan) at E10.5, E12.5 and E18.5, along with adult human cornea samples (SightLife, Seattle, WA, USA) were cryopreserved with the Tissue-Tek® O.C.T Compound (Sakura Finetek USA Inc., Torrance, CA, USA). The frozen sections (10 μm thickness) were mounted on a MembraneSlide NF 1.0 PEN (D) (Carl Zeiss Microscopy GmbH, Göttingen, Germany), and dyed with Cresyl Violet Staining (Sigma-Aldrich, St. Louis, MO, USA). The images of Cresyl Violet Staining samples were obtained using BZ-9000 Fluorescence Microscope (KEYENCE, Osaka, Japan). The monolayers of the corneal epithelium and the basal layer of the oral mucosal epithelium were micro-dissected from the sections of mice embryos using a PALM MicroBeam (Carl Zeiss Microscopy GmbH). The sections of the human corneal epithelium were micro-dissected from four areas; central-apical, central-basal, limbal-apical and limbal-basal cornea. The

conjunctival epithelium was also micro-dissected.

Cell culture

The human corneal cells were incubated with 3.0 U/mL of dispase II (Life Technologies, Carlsbad, CA, USA) for 1 h at 37 °C. After scraping, the epithelial cells were dissociated using TrypLE™ Express (Life Technologies) for 20 min at 37 °C. The dissociated cells were then cultured in Dulbecco's Modified Eagle's Medium (DMEM): F12 Medium (1:1) (Life Technologies) supplemented with B-27® supplement (Life Technologies)、10 μM of Y-27632 (Wako Pure Chemical Industries, Osaka, Japan), 20ng/mL of recombimant human KGF/FGF-7 (R & D Systems, Minneapolis, MN, USA) and 2mM of L-Glutamine (Life Technologies)[42], and incubated in 5 % CO_2 at 37 °C. The immortalized human oral keratinocytes (OKF6/TERT-1 cells and OKF6/TERT-2 cells) and the immortalized human skin keratinocytes (N/TERT-1 cells and N/TERT-2 cells) were obtained from the laboratory of Dr. J. Rheinwald (Harvard Institutes of Medicine, Boston, MA, USA)[19]. The cells were cultured in Keratinocyte-SFM, supplemented with 25 μg/mL of bovine pituitary extract (BPE), 0.2 ng/mL of epidermal growth factor (EGF, Life Technologies) and 0.3 mM of $CaCl_2$ (Wako Pure Chemical Industries). Again, the cells were incubated in 5 % CO_2 at 37 °C. The other human cells which were used in the current study and their culture media are listed in

Supplementary Table S6.

Viral transduction

Two isoforms of PAX6 (NM_000280 and NM_001604), along with their DNA-binding domain truncated mutants (PAX6ΔPAI, PAX6-aΔRED and PAX6-bΔRED) were subcloned into a pLenti7.3/V5-DEST™ Vector (Life Technologies). OCT4, KLF4 and *lacZ* (control) were also subcloned into the pLenti7.3/V5-DEST™ Vector. Each Yamanaka factor (OCT4, SOX2, KLF4, and c-Myc), which had been subcloned into CSV-CMV-MCS-IRES2-Venus plasmids, was kindly provided by Dr. Hiroyuki Miyoshi from the RIKEN BioResource Center. In addition, we constructed all-in-one vectors, which express 'PAX6 and KLF4' or 'PAX6, OCT4 and KLF*4*' simultaneously on pLenti7.3/V5-DEST™ Vector, by using the *2A* sequence[43].

The lentiviruses were produced using the ViraPower™ Lentiviral Expression System (Life Technologies), according to the manufacturer's protocol. Briefly, the expression constructs were co-transfected into 293FT producer cells with an optimized packaging mix and Lipofectamine® 2000. The lentiviral vectors provided by Dr. Miyoshi were co-transfected into 293T cells with pCMV-VZV-G-RSV-Rev, pCAG-HIV-gp (again, kindly provided by Dr. Miyoshi) and FuGene® HD (Roche Applied Science, Manheim, Germany). Twenty-four h following the transfection, the medium was replaced and the cells were cultured for another 48 h. The

virus-containing supernatants were filtered through a 0.45 μm filter (Pall Life Sciences, Ann Arbor, MI, USA), then ultra-centrifuged in an Optima L-90K Preparative Ultracentrifuge (Beckman Coulter, Brea, CA, USA) for 1.5 h at 50000 g, and condensed 10-100 times. The cells were seeded on a 48-well plate 24 h prior to infection. After the viruses and 6 μg/mL of polybrene (Nacalai tesque, Kyoto, Japan) were added to the cells, they were cultured for 24 h, followed by an additional 48 h of incubation in fresh medium. We determined the titer of the lentiviruses with a Global UltraRapid™ Titering Kit (System Biosciences, Mountain View, CA, USA) and also checked the percentage of GFP or Venus-positive cells by fluorescence-activated cell sorting (FACS). The expression levels of the transduced factors were confirmed by quantitative RT-PCR (qRT-PCR).

Immunofluorescence staining

The frozen sections and the cultured cells were fixed in 100 % cold methanol (Wako Pure Chemical Industries) for at least 1 h at -20 °C or 4 % paraformaldehyde (Wako Pure Chemical Industries) for 30 min at room temperature. The fixed samples were blocked and permeabilized with a buffer containing 5 % normal donkey serum (Jackson ImmunoResearch Laboratories, West Grove, PA, USA) and 0.3% Triton X-100 (Sigma-Aldrich) for 1 h at room temperature, and then incubated with a mouse anti-keratin 3/76 (AE5) antibody (1:200,

PROGEN Biotechnik GmbH, Heidelberg, Germany), a goat anti-keratin 12 antibody (1:200, Santa Cruz Biotechnology, Santa Cruz, CA, USA), a rabbit anti-NANOG antibody (1:100, Abcam, Cambridge, UK), a mouse anti-SSEA4 antibody (1:66.7, Abcam), and a mouse anti-TRA-1-60 antibody (1:200, GeneTex, Irvine, CA, USA) overnight at 4 °C. The samples were then incubated with fluorescent-conjugated mouse, goat and rabbit secondary antibodies (Life Technologies) for 1 h at room temperature, and then Hoechst 33342 (DOJINDO, Kumamoto, Japan) was added before the image analysis was performed. The immunofluorescence micrographs and the phase images were taken using an inverted microscope (Axio Observer. D1, Carl Zeiss AG, Oberkochen, Germany) and processed by AxioVision LE (Carl Zeiss Microscopy GmbH). When staining with mouse anti-PAX6 (1:66.7, Santa Cruz Biotechnology) and rabbit anti-KLF4 (1:66.7, Santa Cruz Biotechnology) as the primary antibodies, the fixed cells were permeabilized with a buffer containing 1 % normal donkey serum and 0.3% Triton X-100 for 3 days at 4 °C, followed by blocking with a buffer containing 5 % normal donkey serum and 0.3% Triton X-100 for 1 h at room temperature, and then incubated with the primary antibodies for another 3 days at 4 °C, followed by the incubation with the secondary antibodies.

Quantitative reverse transcription PCR (qRT-PCR)

The total RNA was extracted using the RNeasyPlus Micro Kit (QIAGEN, Hilden, Germany), and cDNA synthesis was performed using the SuperScript® III First-Strand Synthesis System (Life Technologies) with the randomized primer, according to the manufacturer's instructions. The cDNA was subjected to quantitative PCR amplification with the TaqMan® Gene Expression Assay probes (**Supplementary Table S7**) and the Taqman® Fast Universal PCR Master Mix (Life Technologies). The cycling conditions were 95 °C for 20 s and 45 cycles of [95 °C /3 s; 60 °C /30 s]. In addition to the pre-designed primers and probes (Life Technologies) shown in **Supplementary Table S7**, we designed two types of PAX6 primers and probes to distinguish the expression of the two variants of *PAX6* (Forward primer: 5'-GCTCGGTGGTGTCTTTGTCAA-3', Reverse primer: 5'-ACCTGCCCAGAATTTTACTCACA-3', probe: 5'-FAM-TTCTGCAGGTGTCCAAC-MGB-3' for NM_000280 (PAX6-a), Forward primer: 5'-GCTCGGTGGTGTCTTTGTCAA-3', Reverse primer: 5'-TTGTCCAGCACTTGGACTTTTG-3', probe: 5'-FAM-CTGCAGACCCATGCAG-MGB-3' for NM_001604 (PAX6-b)).

For the SYBR green quantitative PCR, the cDNA was subjected to quantitative PCR amplification with the designed primers (**Supplementary Table S8**) and the SYBR Premix Ex Taq™ GC (TaKaRa Bio, Otsu, Japan), according to manufacturer's instructions. The cycling conditions were 95 °C for 30 s and 45 cycles of [95 °C /10 s; 60 °C /30 s]. Melting

curve analyses were performed at the end of the amplification, to confirm the specificity of the amplified products and the lack of primer dimers. The expected lengths of the amplified products were verified using gel electrophoresis. All qRT-PCRs for each sub-group of cells were performed with the RNAs obtained from at least four different samples. The cDNA from the islet cells (Primary Cell Co. , Sapporo, Japan) and the ocular tissues (SightLife), including corneal epithelium, retinal pigment epithelium (RPE), lens and conjunctiva, were used as the positive controls for the qRT-PCR analysis. The final mRNA levels were normalized to the *GAPDH* levels.

Single-cell gene expression analysis

A single-cell gene expression analysis was performed using a Fluidigm Single-Cell Gene Expression Workflow system (Fluidigm, San Francisco, CA, USA). The cells in the trypsinized suspension were mixed with the C_1^{TM} Cell Suspension Reagent, which was then loaded into a prepared C_1 IFC chip. The IFC chip was placed into the C_1^{TM} Single-Cell Auto Prep System, and the cells were divided into single wells. We then performed cell lysis, reverse transcription, and pre-amplification on the C_1^{TM} System, and the reaction products were subjected to the 24 Taqman® assays using the 192.24 chip on the BioMark™ HD System. The Taqman® probes used are listed in **Supplementary Table S7**. The data that were

collected were analyzed with the SINGuLAR^{TM} Analysis Toolset 3.0. The samples with low *18S* expression were removed from the data.

Preparation of the RNA-seq libraries and analysis of the RNA-seq data

Mouse RNA was extracted using an RNeasyPlus Micro Kit, and was amplified using an Arcturus RiboAmp® PLUS KIT (Life Technologies). For each condition, the RNA of four replicates of the transduced OKF6/TERT-1 cell samples was extracted using ISOGEN (Wako Pure Chemical Industries), according to the manufacturer's protocol. The RNA-seq libraries were constructed using the TruSeq RNA Sample Prep kit, v2 (Illumina, San Diego, CA, USA), according to the manufacturer's protocol. Briefly, poly-A containing mRNA was purified with the addition of oligo-dT attached magnetic beads. Following purification, the mRNA was fragmented into small pieces by incubation in a fragmentation buffer at 94 °C for 8 min. The RNA fragments were then copied into first-strand cDNA using random primers and SuperScript® II (Life Technologies). The second-strand cDNA was synthesized using RNaseH and DNA pol I (Illumina). Illumina sequencing adaptors were ligated to the cDNA ends. The cDNA was amplified by 15 PCR cycles using Phusion DNA Polymerase (Finnzymes, Vantaa, Finland). The 101 base pair (bp), paired-end reads were generated using Illumina instruments (HiSeq 2000 for the mouse samples, and HiSeq 2500 for the

human samples).

For the mapping and the quantification of the sequenced reads, we used the TopHat2 (v.2.0.7)/Cufflinks (v.2.0.5) pipeline coupled with Bowtie2 (v.2.0.5)[44]**.** The pipeline, with specific parameters (--read-mismatches 3, --read-edit-dist 3, --b2-sensitive), aligned the reads to the reference genomes (mm9 for mouse samples and hg19 for human samples), and then assembled the RefSeq transcripts for each replicate. We used Cuffcompare to merge all the transcript assemblies. Using the merged assembly, we performed Cuffdiff, which quantifies the RNA abundance, expressed as the number of fragments per kilobase of exon per million mapped reads (FPKM) across all replicates. The differentially expressed genes (>2.0 fold change) were identified with two-group t-tests, coupled with a Benjamini–Hochberg false discovery rate (FDR) procedure by the Cuffdiff. Among 20.9-81.8 million raw reads from the Illumina instruments, this pipeline produced 84-91% of total reads uniquely mapped to the reference genome (**Supplementary Table S9**). The reference genomes and RefSeq annotation were downloaded from the http://genome.ucsc.edu/ database.

Regression promoter modelling

To identify the potential key regulators, we used a linear regression model that was previously proposed[24]**.** Briefly, the linear regression model is as follows:

$$\log Y_i = \sum_j w_j X_{ij} + e_i$$

$$X_{ij} = \sum_k x_k$$

where Y_i is the FPKM of gene *i*, X_{ij} is TFBS-Gene association score (TGAS) of the *j*th TFBS in the promoter region of gene *i*, w_j is the regression coefficient (RC) of the *j*th TFBS, and e_i is the error term. TGAS is the sum of the x_k scores, where *k* represents the position of the *j*th TFBS in the promoter of gene *i*. The score x_k was calculated by

$$x_k = s_k \times L_k \times \left[1 + \sum_n FC_n\right]$$

where *s* is a matrix similarity scored by MATCH[45]**,** *L* is the location-dependent weight[24], FC_n is the fold-change in the expression of the transcription factor *n* that binds to the *j*th TFBS. If FPKM of a transcription factor *n* is ≤ 1.0, then $FC_n = 0$. To search for TFBSs and their associated transcription factors from TRANSFAC professional (released in January 2013)[23], we prepared DNA sequences of +/- 6k bp from the transcriptional start sites (TSSs), and applied the MATCH tool in the minimized false-positive mode, which does not include the KLF4-binding sites. We built a regression model for a set of DUGs, and then reduced the model using AIC (Akaike's Information Criterion). Starting with this reduced model, we repeatedly performed the stepwise selection of the regression model[24], 1000 times for each set of DUGs. The networks (**Supplementary Fig. S3b, e and Fig. 4e**) were visualized using Cytoscape software (www.cytoscape.org).

Dual secreted reporter assay

The dual secreted reporter assay was performed using a Ready-To-Glow™ Dual Secreted Reporter Assay (Clontech Laboratories, Mountain View, CA, USA). We transfected three types of vectors into OKF6/TERT-1 cells at the same time. For the first vector, we used a pSEAP2-control for normalization. We replaced its *SV40* promoter with the *CMV* promoter, because we found that the expression of SEAP was reduced with the *SV40* promoter, but relatively high with the *CMV* promoter, in the OKF6/TERT-1 cells. For the second vector, we sub-cloned various lengths of DNA, 6K bp upstream of *KRT12* into a pMetLuc2-reporter from PAC (RP5-1110E20; BACPAC Resource Center, Oakland, CA, USA); we named these *KRT12-1K*, *KRT12-2K*, *KRT12-3K*, *KRT12-4K*, *KRT12-5K* and *KRT12-6K* reporters, respectively. Similarly, we sub-cloned various length of DNA, 6K bp upstream of *KRT3* into a pMetLuc2-reporter from BAC (RP11-136M20; Empire Genomics, Buffalo, NY, USA), which were termed *KRT3-1K*, *KRT3-2K*, *KRT3-3K*, *KRT3-4K*, *KRT3-5K* and *KRT3-6K* reporters, respectively. Every reporter contained 17 bp of 5'-untranslated region (5'-UTR) of the *KRT12* exon 1 or 64 bp of 5'-UTR of *KRT3* exon 1. For the third vector, we used the pLenti7.3/V5-DEST™ Vector for the overexpression of PAX6, OCT4, KLF4, lacZ and their combinations. The OKF6/TERT-1 cells were seeded at a concentration of 3.0×10^4 cells per 96-well plate

24 h before transfection. The three types of vectors, i.e. a pSEAP2-control, one of the pMetLuc2-reporters and one of the pLenti7.3/V5-DEST^{TM} vectors, were co-transfected using the Lipofectamine® 3000 reagent (Life Technologies). The culture medium was collected 24 h following transfection, after which a SEAP assay and a secreted metridia luciferase assay were performed, following the manufacturer's recommended procedure. The signal (luminescence) of the secreted metridia luciferase was compensated by the signal of SEAP in each sample. The obtained data was compared to the results from the samples which were transfected with lacZ subcloned pLenti7.3/V5-DEST^{TM}.

Co-immunoprecipitation (Co-IP) and mass spectrometry (MS)

We used an EpiXplore^{TM} Nuclear Co-Immunoprecipitation Kit (Clontech Laboratories) to identify the protein-protein complexes. The nuclear extracts were incubated with a rabbit anti-PAX6 antibody (1:50) for 24 h. The purified proteins were subjected to sodium dodecyl sulfate poly-acrylamide gel electrophoresis (SDS-PAGE), followed by extraction of the target lanes and a liquid chromatography-mass spectrometry (LC-MS/MS) analysis. The LC-MS/MS analysis was conducted using a UltiMate® 3000 Nano LC system (Thermo Fisher Scientific, Waltham, MA, USA), coupled to a Q-Exactive^{TM} hybrid quadrupole-Orbitrap mass spectrometer (Thermo Fisher Scientific) with a nano-electrospray ionization source. The raw

data files were analyzed with the Mascot Distiller software, v2.2 (Matrix Science, London, UK), to create peak lists based on the recorded fragmentation spectra. The peptides and proteins were identified with the Mascot software¸v2.3 (Matrix Science), against a UniPort database with a precursor mass tolerance of 10 ppm, an ion mass tolerance of 0.01 Da, and a strict trypsin specificity allowing for up to two missed cleavage sites. Carbamidomethylation of the cysteine residues was set as a fixed modification, whereas the oxidation of methionine residues was allowed as a variable modification.

Tetracycline-on (Tet-On) system

We generated a ViraPower™ T-Rex™ OKF6/TERT-1 cell line with the transduction of pLenti3.3/TR (Life Technologies). All-in-one cassettes, which include PAX6 (PAX6-a or PAX6-b), OCT4 and KLF4, were sub-cloned into the pLenti6.3/TO/V5-DEST vector (Life Technologies). After we transduced a ViraPower™ T-Rex™ OKF6/TERT-1 cell line with the pLenti6.3/TO/V5-DEST vectors, we selected four PAX6-a-OCT4-KLF4- and PAX6-b-OCT4-KLF4-inducible colonies with Blasticidin (Life Technologies) selection. Thus, we established the tetracycline on (Tet-On) system for the controlled expression of PAX6-a-OCT4-KLF4 and PAX6-b-OCT4-KLF4.

Treatment with small molecules

Some samples were subjected to a treatment with small molecules during 24 h of viral infection and the following 48 h. The small molecules used in these experiments were 600 nM of 6-Bromoindirubin-3'-oxime (BIO, Wako Pure Chemical Industries), 3 μM of BIX01294 (BIX, Stemgent, San Diego, CA, USA), 120 nM of RG108 (Stemgent), 6 μM of R(+)BayK 8644 (BayK, Stemgent) and 300 μM of Valproic Acid (VPA, Stemgent). The optimal concentrations of these molecules were determined in preliminary experiments, and the maximum concentrations that did not show any apparent toxicity were selected.

Bioinformatics analysis

The enrichment analysis of Gene Ontology (GO) biological process terms was performed with the GOFunction software package of Bioconductor (v.3.0, http://www.bioconductor.org/) with a Bonferroni *p*-value correction (< 0.01). The R programming language (http://www.r-project.org/) was used for the regression modelling. The statistical significance of the set of 1000 RCs was tested with a one-sample t-test after Bonferroni correction (< 0.01 *p*-value). All the other statistical analyses were performed using the JMP® Pro software, version 10.0.0 (SAS Institute, Cary, NC, USA). A t-test was used to evaluate the difference between the two groups. A two-sided Dunnett’s test and a paired t-test with a *Bonferroni correction* were used

as the multiple comparisons. A correlation coefficient (r) was applied to the calculation of the correlation between two genes, and a *p*-value < 0.05 was considered statistically significant.
